# Unsupervised Tree Boosting for Learning Probability Distributions

**Published:** 2024

**Authors:** Naoki Awaya, Li Ma

**Affiliations:** School of Political Science and Economics, Waseda University, Shinjuku City, Tokyo 169-8050, Japan; Department of Statistical Science, Duke University, Durham, NC 27708, USA

**Keywords:** generative models, normalizing flows, additive models, density estimation, ensemble methods, recursive partitioning

## Abstract

We propose an unsupervised tree boosting algorithm for inferring the underlying sampling distribution of an i.i.d. sample based on fitting additive tree ensembles in a manner analogous to supervised tree boosting. Integral to the algorithm is a new notion of “addition” on probability distributions that leads to a coherent notion of “residualization”, i.e., subtracting a probability distribution from an observation to remove the distributional structure from the sampling distribution of the latter. We show that these notions arise naturally for univariate distributions through cumulative distribution function (CDF) transforms and compositions due to several “group-like” properties of univariate CDFs. While the traditional multivariate CDF does not preserve these properties, a new definition of multivariate CDF can restore these properties, thereby allowing the notions of “addition” and “residualization” to be formulated for multivariate settings as well. This then gives rise to the unsupervised boosting algorithm based on forward-stagewise fitting of an additive tree ensemble, which sequentially reduces the Kullback-Leibler divergence from the truth. The algorithm allows analytic evaluation of the fitted density and outputs a generative model that can be readily sampled from. We enhance the algorithm with scale-dependent shrinkage and a two-stage strategy that separately fits the marginals and the copula. The algorithm then performs competitively with state-of-the-art deep-learning approaches in multivariate density estimation on multiple benchmark data sets.

## 1. Introduction

In supervised learning such as classification and regression, boosting is acknowledged as one of the most powerful algorithms. It is acclaimed for the ability to overcome the curse of dimensionality and achieve a desirable balance in the bias-variance trade-off. The most popular boosting algorithms can be thought of as sequentially fitting an additive ensemble of weak learners, often in the form of regression or classification trees (e.g., Friedman, 2001; Hastie et al., 2009). The success of tree boosting in supervised problems suggests that a similar strategy might also prevail in unsupervised problems, where the ultimate objective involves learning the structures of some unknown probability distribution based on a collection of training data from that distribution.

Our aim in this paper is to formulate a new additive tree model framework for probability distributions along with an unsupervised boosting algorithm that inherits the strength of the supervised boosting. Our approach is motivated by the observation that some highly effective supervised tree boosting algorithms are fitted in each iteration based on a set of residuals rather than the original observations, thereby substantially simplifying the optimization task in each iteration. To realize this strategy in the unsupervised context, we introduce a notion of *addition* specialized for probability measures which leads to a natural concept of the *residual* of an observation after “subtracting” a probability measure from it. It should be clarified that in the unsupervised setting, the natural operation of addition—such as through taking weighted averages—does not work, as it is not straightforward to find such an embedding that renders a conceptually and computationally simple notion of “residualization” of an observation, which removes a fitted measure from the underlying sampling distribution.

The notions of “addition” and “residualization” for probability distributions are formulated in terms of cumulative distribution function (CDF) transforms and compositions. We start from the case of univariate distributions, for which the addition of two measures can be defined simply in terms of a composition of their CDFs whereas the residual of an observation from subtracting a measure is simply the application of the corresponding CDF transform to that observation. In generalizing this notion of addition to multivariate distributions on $R^{d}$ with d>1, however, the classical notion of the multivariate CDF, which maps $R^{d}$ to the interval (0, 1] is unsatisfactory, as easily seen, for example, by the fact that one can neither define a composition of two such CDFs nor define residuals that still lie in $R^{d}$. More fundamentally, multivariate CDFs do not preserve a set of “group-like” properties of 1D CDFs that underlie the notion of addition and residualization. Interestingly, it can be shown that a proper notion of the CDF for multivariate distributions, which maps $R^{d}$ to $R^{d}$, does exist for probability measures defined on tree-partition structures (tree measures), and it naturally generalizes the notions of addition and residuals to multivariate settings.

Based on these notions, we introduce an unsupervised tree boosting algorithm for learning probability measures based on forward-stagewise (FS) fitting of an additive tree ensemble. Our algorithm in each iteration completes two operations that resemble those in supervised boosting: (i) computing the current residuals by subtracting the fitted measure at the current iteration from the observations and (ii) fitting a tree-based weak learner on the residuals and adding the estimated distribution to the current fit. The algorithm enables straightforward analytical evaluation of the probability density of the fitted distribution and produces a generative model for the fitted measure that can be directly sampled from.

Because the notion of addition on probability measures in our boosting framework takes the form of function compositions, one can also view our approach as a normalizing flow (NF) (see i.e., Papamakarios, 2019; Papamakarios et al., 2021; Kobyzev et al., 2020). In NF approaches, one seeks to find a sequence of transformations that moves the observed distribution into a baseline distribution such as a uniform or a Gaussian. Most notably, Inouye and Ravikumar (2018) introduced a tree-based NF method through composing a class of transforms that are essentially equivalent to the generalized CDF transforms we introduce. As such, their NF algorithm—called “tree density destructors” is in essence the same as our boosting algorithm aside from the difference in the choice of the base learner, the strategies in regularization, and the other boosting-inspired specification strategies. These different practical choices we make—which are largely motivated from the boosting perspective—do lead to substantial differences in empirical performance and computational efficiency, as we will demonstrate in our numerical experiments. Besides, formulating the algorithm from the boosting perspective also allows us to provide a more rigorous theoretical grounding for the algorithm. Therefore, our main contribution is not in the novelty of the algorithm itself, but in connecting boosting and NF in both theory and practice. In summary, our contributions are:

1. *Reformulation of tree-based NFs as tree boosting*. We introduce a formal notion of addition on probability measures that leads to the group structure on (generalized) CDF transforms, based on which we show that tree-based NFs can be understood as iterative fitting of an additive ensemble of probability measures in a manner analogous to iterative fitting of weak learners to residuals under classical tree boosting for supervised problems.
2. *Theoretical justification*. The boosting formulation allows us to justify the iterative algorithm formally from a decision-theoretic perspective in terms of sequential minimization of the Kullback-Leibler (KL) divergence between the model and the unknown true measure. In addition, analogous to supervised tree boosting (Breiman, 2004), we show that the unsupervised tree ensemble is “highly expressive” in the sense that a wide class of distributions can be represented or well approximated by a finite combination of highly constrained (or “weak”) tree-based density models.
3. *Methodological improvement*. The boosting formulation allows us to design and incorporate methodological techniques—inspired by techniques originally developed for supervised boosting. These include guidelines for choosing the number of trees, setting the appropriate level of shrinkage/regularization, and choosing/specifying the base learner. We provide a comprehensive empirical evaluation of our proposed boosting algorithm by comparing it with the density destructor (Inouye and Ravikumar, 2018) and other state-of-the-art NF algorithms such as Masked Autoregressive Flow (MAF) (Papamakarios et al., 2017) using simulation examples and benchmark data sets. The results suggest that our new algorithm substantially improves performance over tree density destructors and is competitive with the state-of-the-art deep-learning based NF algorithms at a substantially smaller computation cost. The decision-theoretic formulation also leads to a natural measure of variable importance based on the respective contribution of each dimension in reducing the overall KL divergence, which provides additional insights into the relevance of each dimension in characterizing the underlying distribution and allows effective variable screening.

We note that boosting for unsupervised learning has been considered by Ridgeway (2002); Rosset and Segal (2002); Cui et al. (2021). These previous attempts however aim at constructing an ensemble in the form of a weighted average of probability measures and fit such ensembles through gradient boosting (Mason et al., 1999) under various loss functions. These unsupervised gradient boosting algorithms have yet to be demonstrated to be computationally efficient or to perform well in high-dimensional continuous sample spaces.

All proofs are given in Appendices A and B.

## 2. Method

In this section, we start by defining notions of addition and residuals for one-dimensional settings and then generalize them for multivariate distributions to introduce the new tree boosting algorithm.

### 2.1 CDF-based Addition and Residualization for Univariate Distributions

Without loss of generality, let (0, 1] represent the one-dimensional sample space. For ease of exploration, we shall assume that the distributions are absolutely continuous on the sample space with respect to the Lebesgue measure and have full support.

We first make an observation that if a random variable X~G, its sampling distribution, then G(X)~Unif(0,1], where G denotes the CDF of G. (Throughout we will use bold font letters to indicate the CDFs of the corresponding distributions.) As such, the CDF transform “removes” the distributional structure of G from the sampling distribution of X. Thus one can think of r=G(X) as a “residual”. Moreover, Unif(0,1] serves as the notion of “zero”, whose CDF is the identity map, in the space of probability distributions as it is the remaining distribution after “subtracting” the true sampling distribution G from X.

Next we define a notion of “adding” two distributions G and H that is consistent with the above notion of “subtraction” or “residualization”. Specifically, the addition of $G_{1}$ and $G_{2}$, denoted as $“G_{1}⊕G_{2}”$, should satisfy the property that if $X\sim G_{1}⊕G_{2}$, then $r^{\left(1\right)}=G_{1}(X)\sim G_{2}$. In other words, if the sampling distribution of X is the “sum” of $G_{1}$ and $G_{2}$, then taking the “residual” of X with respect to $G_{1}$ should result in a random variable distributed as $G_{2}$. Such a notion of addition indeed exists:

$$G_{1}⊕G_{2}\;\text{is the distribution whose CDF is}\;G_{2}\circ G_{1}$$

where “∘” denotes function composition. Note that this notion of addition is not commutative. That is, $G_{1}⊕G_{2}\neq G_{2}⊕G_{1}$. Fortunately, as we will see, the operation of fitting an additive ensemble as in supervised boosting requires only a non-abelian group structure, which does not require the commutativeness of the underlying addition. As such, the loss of commutativeness will pose no difficulty in our construction of additive tree models and later an unsupervised boosting algorithm based on the new notions of addition and residuals.

By iteratively applying such an addition, one can define the “sum” of k(≥1) probability measures $G_{1},\ldots ,G_{k}$. Specifically, the sum of $G_{1},\ldots ,G_{k}$,

$$G_{1}⊕\cdots ⊕G_{k}\;\text{is the distribution whose CDF is}\;G_{k}\circ \cdots \circ G_{1}$$

The following property provides the basis for sequential addition and residualization, analogous to those in supervised boosting.

**Proposition 1.** *If* $G_{1},\ldots ,G_{k}$ *have full support on* (0,1], (*i.e., when the CDFs* $G_{1},\ldots ,G_{k}$ *are strictly increasing*), *then for any* i=1,2,…,k-1

$$X\sim G_{1}⊕\cdots ⊕G_{k}\;\text{if and only if}\;r^{\left(i\right)}=G_{i}\circ \cdots \circ G_{1}(X)\sim G_{i+1}⊕\cdots ⊕G_{k}.$$

Proposition 1 then implies that such residualization can be applied sequentially. That is, if $r^{\left(k\right)}$ is the residual of x after subtracting $G_{1}⊕\cdots ⊕G_{k}$, then $r^{\left(0\right)}=x$, and for k≥1

(1) $$r^{\left(k\right)}=G_{k}\left(r^{\left(k-1\right)}\right)=G_{k}\circ G_{k-1}\left(r^{\left(k-2\right)}\right)\cdots =G_{k}\circ G_{k-1}\circ \cdots \circ G_{1}\left(r^{\left(0\right)}\right).$$

The “additivity” induced by the composition of CDFs also induces an additivity on the corresponding log-likelihood. Specifically, suppose $g_{i}=dG_{i}/d\mu$ is the probability density function (pdf) of $G_{i}$ for i=1,2,…,k with respect to Lebesgue measure μ. Then the density of the ensemble measure $F_{k}≔G_{1}⊕\cdots ⊕G_{k},f_{k}=dF_{k}/d\mu$, satisfies

$$f_{k}\left(x\right)=\prod_{i=1}^{k}g_{i}\left(r^{\left(i-1\right)}\right)\;\text{or}\;\text{log}\;f_{k}\left(x\right)=\sum_{i=1}^{k}\text{log}\;g_{i}\left(r^{\left(i-1\right)}\right).$$

Table 1 summarizes the corresponding notions of addition, residuals, and zero in supervised boosting (in particular regression) and those in our unsupervised formulation. With these new notions, we are ready to introduce a boosting algorithm for learning one-dimensional distributions. However, the more interesting application involves multivariate (in fact high-dimensional) distributions. As such, we first generalize these notions to multivariate cases and then introduce a multivariate version of our boosting algorithm that contains the (less interesting) univariate scenario as a special case.

### 2.2 Generalization to Multivariate Distributions

The above notions of addition and residuals do not find direct counterparts for multivariate measures if one uses the traditional definition of CDFs for multivariate distributions. In particular, because the traditional CDF is a mapping from $(0,1{]}^{d}$ to (0, 1] instead of $(0,1{]}^{d}$, we cannot even take the composition of the CDFs or compute the residuals, which should remain in the same space as the original observations. Beyond the minimal requirement that the appropriate notion of “CDF” should map from $(0,1{]}^{d}$ to $(0,1{]}^{d}$, it must also enjoy several group-like properties of univariate CDF’s.

We summarize four such properties that the “CDF” must satisfy to allow the definition of addition and residualization to carry over into the multivariate setting:

(C1) G is a mapping from $(0,1{]}^{d}$ to $(0,1{]}^{d}$.

(C2) G is uniquely determined by G.

(C3) If X~G, then $G(X)\sim \text{Unif}\left((0,1{]}^{d}\right)$, the “zero”.

(C4) If $X\sim G_{1}⊕G_{2}$, the distribution is uniquely determined by its “CDF” $G_{2}\circ G_{1}$, then $G_{1}(X)\sim G_{2}$.

Remark: (C1) and (C2) are needed for defining addition in terms of compositions. (C3) and (C4) are needed for the proper notion of residuals.

For the purpose of constructing a tree additive ensemble model and a boosting algorithm, one type of “CDFs” that satisfy these conditions is particularly useful as it is very easy to compute for probability distributions with piecewise constant densities defined on leaves of a recursive dyadic partitioning of the sample space. As one can imagine, efficient computation of the “CDFs” for tree-based models is critical as they will be computed many times during the fitting to an additive ensemble.

#### 2.2.1 Characterizing probability measures on a recursive dyadic partition tree

Next we describe the construction of this generalized notion of multivariate CDFs, which we call the “tree-CDF”, due to its connection to recursive bifurcating partition trees. We start by introducing some additional notation related to recursive dyadic partitions.

A recursive dyadic partition of depth R is a sequence of nested dyadic partitions ${𝒜}^{1},{𝒜}^{2},\ldots ,{𝒜}^{R}$ on the sample space Ω. The first partition ${𝒜}^{1}$ only includes Ω, and for k=2,…,R, the partition ${𝒜}^{k}$ consists of all the sets generated by dividing each $A\in {𝒜}^{k-1}$ into two children $A_{l}$ and $A_{r}$, where $A_{l}\cup A_{r}=A$ and $A_{l}\cap A_{r}=\emptyset$. (Throughout, we use subscripts l and r to indicate left and right children respectively.) We can denote the recursive partition using a tree $T=\cup_{k=1}^{R}{𝒜}^{k}$. As such, we refer to the sets in the partitions as “nodes”. We call the collection of nodes in ${𝒜}^{R}$ the “terminal” nodes or “leaves” of T and denote it by ℒ(T); the nodes in other levels are the “non-leaf” nodes or “interior” nodes, which we denote by 𝒩(T)=T∖ℒ(T).

We consider partition trees with axis-aligned partition lines. In this case, a node A∈T is of the following rectangular form

(2) $$A=\left(a_{1},b_{1}\right]\times \cdots \times \left(a_{d},b_{d}\right].$$

For a non-leaf node A∈𝒩(T), the children $A_{l}$ and $A_{r}$ are generated by dividing A in one of the d dimensions, say $j^{*}$,

(3) $$A_{l}=\left(a_{1},b_{1}\right]\times \cdots \left(a_{j^{*}},c_{j^{*}}\right]\times \cdots \left(a_{d},b_{d}\right]\;\text{and}\;A_{r}=\left(a_{1},b_{1}\right]\times \cdots \left(c_{j^{*}},b_{j^{*}}\right]\times \cdots \left(a_{d},b_{d}\right].$$

In the following, for each partition tree T, we let ${𝒫}_{T}$ be the class of probability measures that are conditionally uniform on the leaves of T and have full support on Ω. That is,

$${𝒫}_{T}=\left\{G:G\;\text{has full support on}\;(0,1{]}^{d}\;\text{and}\;G(\cdot |A)=\mu (\cdot |A)\;\text{for every}\;A\in ℒ(T)\right\},$$

where μ is the uniform distribution, and G(⋅∣A) and μ(⋅∣A) are the corresponding conditional distributions on A.

To generalize the CDF transform from univariate to multivariate cases, first we note an interesting multi-scale decomposition of the univariate CDF—a univariate CDF transform G for any distribution $G\in {𝒫}_{T}$ on an observation x can actually be computed sequentially in a fine-to-coarse fashion along the branch in the partition tree T in which x falls. Specifically, suppose T has depth R, then for x∈(0,1], let ${\left\{A_{k}\right\}}_{k=1}^{R}$ be a sequence of nodes in T such that $A_{k}\in {𝒜}^{k}$ and

$$x\in A_{R}\subset A_{R-1}\subset \cdots \subset A_{1}=\left(0,1\right].$$

The CDF transform G(x) can be decomposed into the composition of a sequence of “local move functions”

(4) $$G\left(x\right)=G_{A_{1}}\circ \cdots \circ G_{A_{R-1}}\left(x\right),$$

where for any A=(a,b]∈𝒩(T) with two children $A_{l}=(a,c]$ and $A_{r}=(c,b]$, the mapping $G_{A}:A\to A$ is (up to a normalizing constant μ(A)) the CDF of a dyadic piecewise constant density equal to $G\left(A_{l}|A\right)/\mu \left(A_{l}\right)$ on $A_{l}$ and $G\left(A_{r}|A\right)/\mu \left(A_{r}\right)$ on $A_{r}$. More precisely, $G_{A}:A\to A$ is given by

$$\frac{G_{A}(x)-a}{x-a}=\frac{G\left(A_{l}|A\right)}{\mu \left(A_{l}|A\right)}\;\text{for}\;x\in A_{l}\;\text{and}\;\frac{b-G_{A}\left(x\right)}{b-x}=\frac{G\left(A_{r}|A\right)}{\mu \left(A_{r}|A\right)}\;\text{for}\;x\in A_{r}.$$

Note that the conditional measures and the input and output of $G_{A}$ have the following relationship

$$G\left(A_{l}|A\right)>\mu \left(A_{l}|A\right)\;\Leftrightarrow \;G\left(A_{r}|A\right)<\mu \left(A_{r}|A\right)\;\Leftrightarrow \;G_{A}\left(x\right)>x,$$

$$G\left(A_{l}|A\right)<\mu \left(A_{l}|A\right)\;\Leftrightarrow \;G\left(A_{r}|A\right)>\mu \left(A_{r}|A\right)\;\Leftrightarrow \;G_{A}\left(x\right)<x.$$

**Figure 1:**
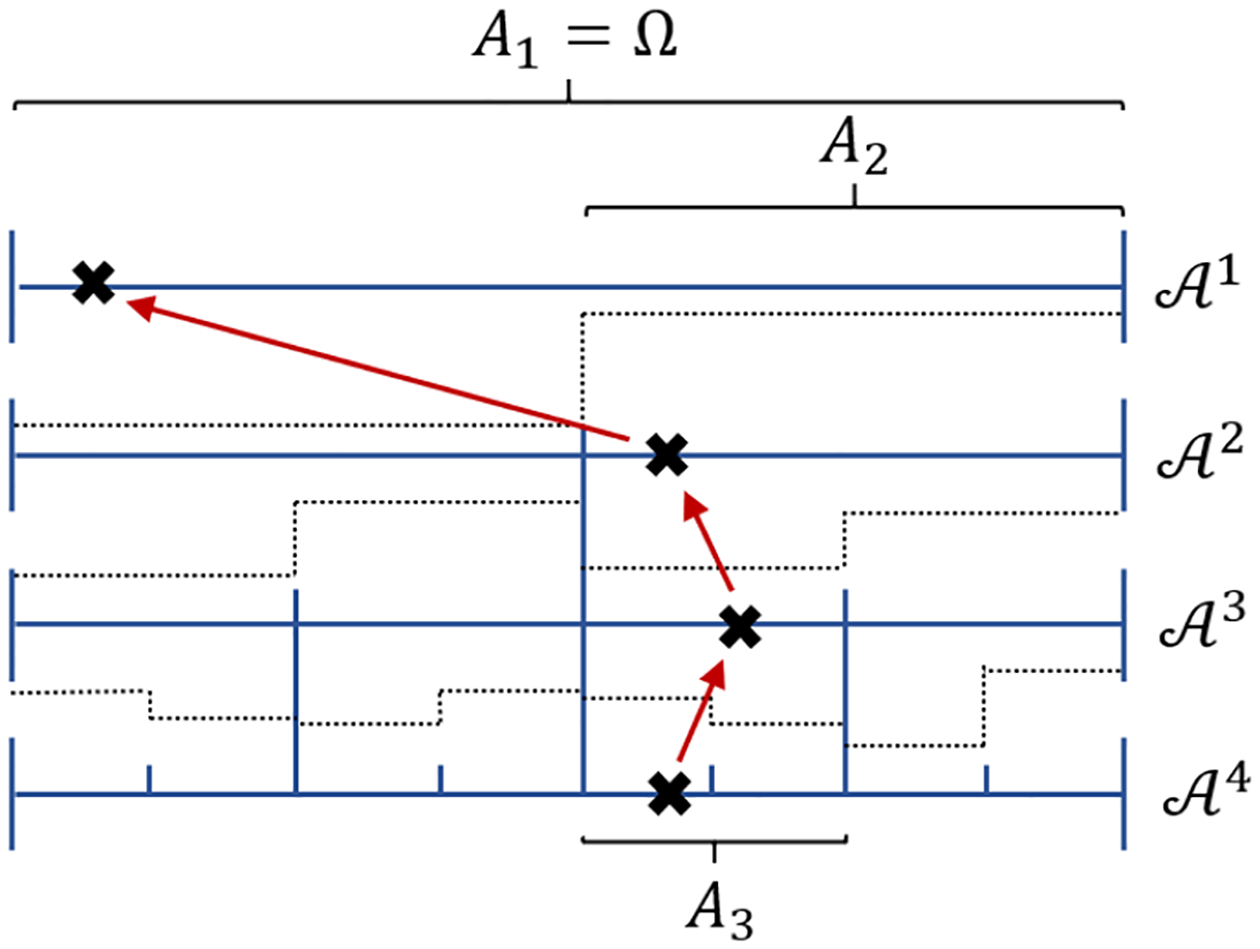
Visualization of the tree-based decomposition of a univariate CDF into three local moves. The dotted lines indicate $G\left(A_{l}|A\right)/\mu \left(A_{l}|A\right)$ or $G\left(A_{r}|A\right)/\mu \left(A_{r}|A\right)$ on each node A.

We call $G_{A}$ a “local move” function because it moves a point in A in the direction of the child node with less (conditional) probability mass than the (conditional) uniform measure as illustrated in Figure 1. The amount of movement on A is proportional to the probability mass differential between the two children of A in G relative to μ.

If we think of applying the univariate CDF transform as “subtracting” the information contained in a probability measure from an observation, the decomposition in Equation 4 indicates that such subtraction can be done sequentially through the local moves, each subtracting a piece of information regarding the measure from the observation. This perspective leads to a generalization of the CDF transform for the multivariate case as we describe below.

For a point $x=\left(x_{1},\ldots ,x_{d}\right)\in \Omega =(0,1{]}^{d}$, again let T be a recursive dyadic partition tree of depth R on the sample space Ω, and ${\left\{A_{k}\right\}}_{k=1}^{R}$ the sequence of nodes in T that contains x as before. Then we define a mapping $G:(0,1{]}^{d}\to (0,1{]}^{d}$ in terms of a sequence of fine-to-coarse local moves along that branch in T. Specifically, for a node A∈𝒩(T) as in Equation 2 with children $A_{l}$ and $A_{r}$ attained from dividing A in the $j^{*}$ th dimension as described in Equation 3, we define a local move mapping $G_{A}:A\to A$ such that for any $x\in A,G_{A}(x)=\left(G_{A,1}(x),\ldots ,G_{A,d}(x)\right)$ where $G_{A,j}(x)=x$ for all $j\neq j^{*}$, and

$$\frac{G_{A,j^{*}}(x)-a_{j^{*}}}{x_{j^{*}}-a_{j^{*}}}=\frac{G\left(A_{l}|A\right)}{\mu \left(A_{l}|A\right)}\;\text{for}\;x\in A_{l}\;\text{and}\;\frac{b_{j^{*}}-G_{A,j^{*}}\left(x\right)}{b_{j^{*}}-x_{j^{*}}}=\frac{G\left(A_{r}|A\right)}{\mu \left(A_{r}|A\right)}\;\text{for}\;x\in A_{r}.$$

**Figure 2:**
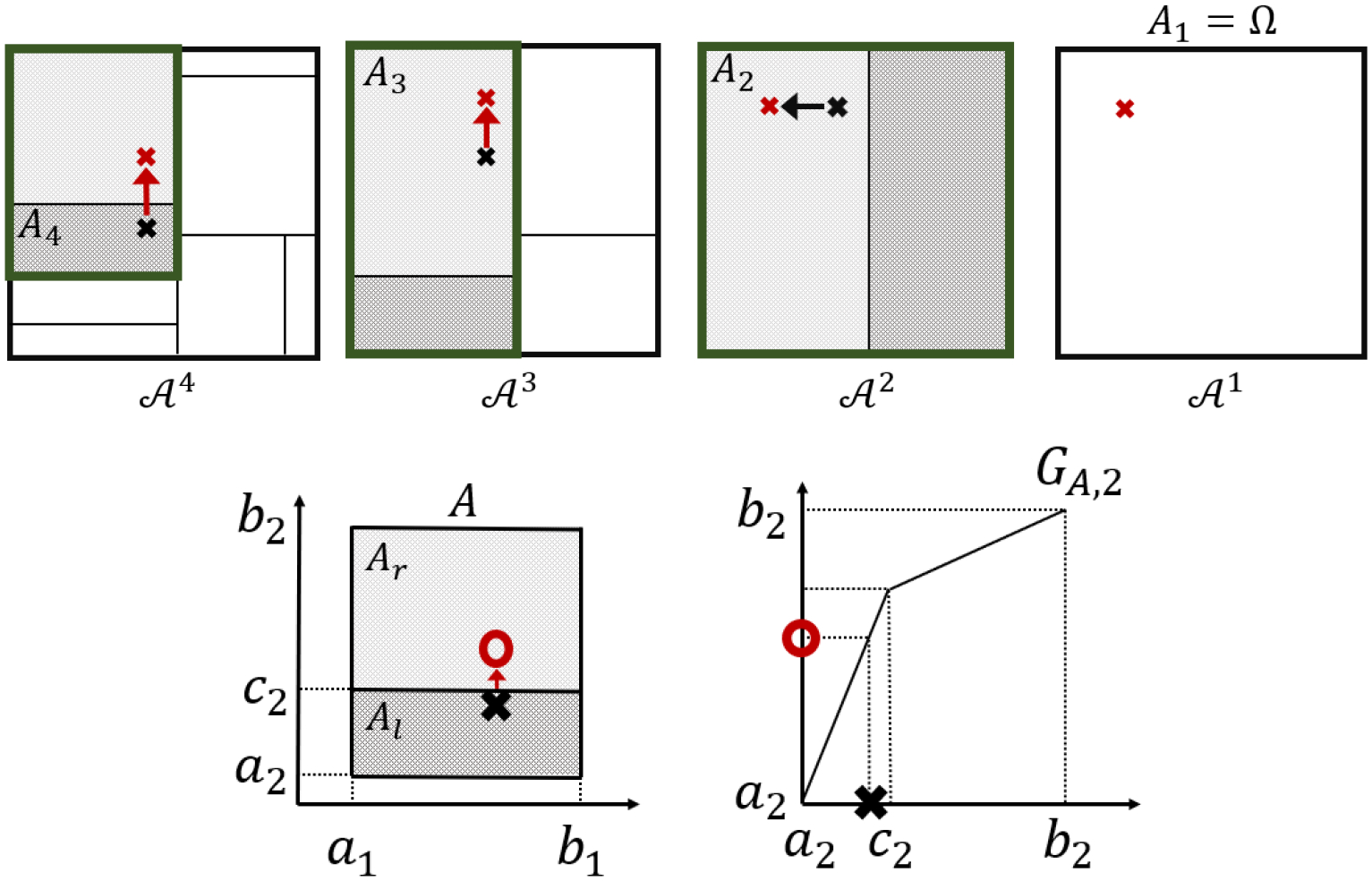
Top: Visualization of the local move functions in each level under R=3. The nodes with the darker color have higher conditional probabilities relative to the uniform measure. Bottom: An example of $G_{A}$ with $j^{*}=2$. The input and the output of $G_{A}$ are indicated by × and ∘, respectively.

As illustrated in Figure 2, similar to the univariate case, the local move mapping is nothing but (up to a normalizing constant μ(A)) the CDF of a dyadic piecewise constant density on A, except that now in the multivariate setting there are a total of d directions in which such a dyadic split can take place. As a transform, it moves x in the direction of the child node with less probability mass relative to the uniform measure.

As before, we now define a mapping $G:(0,1{]}^{d}\to (0,1{]}^{d}$, called a “tree-CDF”, as the composition of these local move functions. That is,

$$G\left(x\right)=G_{A_{1}}\circ \cdots \circ G_{A_{R-1}}\left(x\right).$$

G is injective from $(0,1{]}^{d}$ to $(0,1{]}^{d}$ for any G with full support on $(0,1{]}^{d}$. Additionally, because $G_{A}$ is surjective for every A,G is also surjective. One can also show that G is measurable. Hence we have the following proposition that establishes Condition (C1) for tree-CDFs, which is essential to defining the addition of multivariate distributions in terms of tree-CDF compositions.

**Proposition 2.** *The tree-CDF mapping* $G:(0,1{]}^{d}\mapsto (0,1{]}^{d}$ *is bijective and measurable for any* $G\in {𝒫}_{T}$.

The next two theorems show that our construction of G satisfies Conditions (C2) and (C3) as well. That is, G uniquely determines G, and applying the G mapping to an observation effectively “subtracts” the distributional structure in G from the sampling distribution of that observation.

**Theorem 1.** *A measure* $G\in {𝒫}_{T}$ *for some partition tree* T *can be determined by the tree-CDF mapping* G *as follows*

G(B)=μ({G(x):x∈B})for allB∈ℬ(Ω).

Remark: Theorem 1 establishes (C2) and implies that G uniquely determines G, regardless of the tree based on which G is defined. However, G is tree-specific, that is, to uniquely determine G we need a pair of the measure G and the finite tree T.

**Theorem 2.** *If* $X\sim G\in {𝒫}_{T}$, *then* $G(X)\sim \text{Unif}\left((0,1{]}^{d}\right)$. *Conversely*, *if* $U\sim \text{Unif}\left((0,1{]}^{d}\right)$, *then* $G^{-1}(U)\sim G$.

Remark: Theorem 2 establishes (C3) and shows that if one can compute the inverse map $G^{-1}$ then one essentially has a generative model, which allows generating samples from G based on “inverse-CDF” sampling. More details on this will be given in Section 2.3.

#### 2.2.2 Addition and Residualization for multivariate Settings

Let $G_{1},\ldots ,G_{k}$ be a collection of probability measures such that $G_{l}\in {𝒫}_{T_{l}}$ for l=1,2,…,k, and let $G_{1},\ldots ,G_{k}$ be the corresponding tree-CDFs. As a generalization to the univariate case, next we define addition of distributions by composing their tree-CDFs. We first show that such a composition indeed pins down a unique probability measure.

**Lemma 1.** *For* $G_{l}\in {𝒫}_{T_{l}}(l=1,2,\ldots ,k)$, *the mapping $F_{k}:ℬ(\Omega )\mapsto (0,1]$ defined as*

(5) $$F_{k}\left(B\right)=\mu \left(\left\{G_{k}\circ \cdots \circ G_{1}\left(x\right):x\in B\right\}\right)\;\text{for}\;B\in ℬ\left(\Omega \right).$$

*is a probability measure*.

Now we can define the sum of k distributions, $G_{1}⊕\cdots ⊕G_{k}$, as the measure $F_{k}$ given in Equation 5. This definition of addition contains the univariate case presented earlier as a special case. We note that the addition implicitly involves the tree structures $T_{1},\ldots ,T_{k}$. This dependency on the trees, however, is suppressed in the “ ⊕” notation for simplicity without causing confusion.

Next we turn to the notion of residuals and generalize Proposition 1 to multivariate distributions, which establishes Condition (C4) for tree-CDFs.

**Proposition 3.** *Let* $G_{1},\ldots ,G_{k}$ *be a collection of probability measures such that* $G_{l}\in {𝒫}_{T_{l}}$ *for* l=1,2,…,k. *Then*

$$X\sim G_{1}⊕\cdots ⊕G_{k}\;\text{if and only if}\;r^{\left(i\right)}=G_{i}\circ \cdots \circ G_{1}(X)\sim G_{i+1}⊕\cdots ⊕G_{k}$$

*for any* i=1,2,…,k-1.

Remark: This proposition implies Condition (C4) by setting k=2 and i=1.

Moreover, the sequential update of the residuals given in Equation 1 remains valid. The only difference is that now the residualization in each step depends on an implicit partition tree structure, encapsulated in the corresponding tree-CDF.

### 2.3 Unsupervised Boosting based on Forward-stagewise (FS) Fitting

Equipped with the new notions of addition and residuals, we are ready to generalize our unsupervised boosting algorithm to the multivariate setting based on forward-stagewise (FS) fitting. Suppose we have an i.i.d. sample $x_{1},\ldots ,x_{n}$ from an unknown distribution F, which we model as an additive ensemble of K probability measures

(6) $$F=G_{1}⊕\cdots ⊕G_{K}$$

where each $G_{k}$ is modeled as a member in ${𝒫}_{T_{k}}$ for some (unknown) $T_{k}$. We compute the residuals step-by-step and at the kth step, fit $G_{k}$ to the current residuals. The fit at the kth step produces an estimate for $G_{k}$ and a partition tree $T_{k}$, which are used to define the tree-CDF in the next step for updating the residuals. The algorithm is summarized below.

**Table:** 

**Initialization**
Set $r^{\left(0\right)}=\left(x_{1},\ldots ,x_{n}\right)$.
**Forward-stagewise fitting**
Repeat the following steps for =1,…,K: Fit a weak learner that produces a pair of outputs $\left(G_{k},T_{k}\right)$ to the residualized observations $r^{\left(k-1\right)}$, where $T_{k}$ is an inferred partition tree and $G_{k}$ is the tree-CDF for a measure $G_{k}\in {𝒫}_{T_{k}}$. Update the residuals $r^{\left(k\right)}=\left(r_{1}^{\left(k\right)},\ldots ,r_{n}^{\left(k\right)}\right)$, where $r_{i}^{\left(k\right)}=G_{k}\left(r_{i}^{\left(k-1\right)}\right)$.

The output of the boosting algorithm in terms of the collection of pairs $\left(G_{k},T_{k}\right)$ for k=1,2,…,K contains all of the information from the data regarding the underlying distribution. (In fact the $G_{k}$’s alone contain all the relevant information, but the $T_{k}$’s are indispensable for effectively representing and storing the $G_{k}$’s.)

Next we demonstrate two ways to extract such information. In particular, we show (i) how to compute the density function of the fitted measure F at any point in the sample space analytically, and (ii) how to use the resulting generative model to draw Monte Carlo samples from the fitted measure F based on “inverse-CDF” sampling.

#### 2.3.1 Evaluating the Density Function of F.

Density estimation is a common objective in learning multivariate distributions. The next proposition generalizes the additive decomposition of the log-likelihood for the univariate case and provides a recipe for evaluating the density for the fitted measure F analytically based on the output of the FS algorithm.

**Proposition 4.** *For any* $x\in (0,1{)}^{d}$, *the density* f=dF/dμ *for* F *of the additive form in* Equation 6 *is given as follows*

$$f(x)=\prod_{k=1}^{K}g_{k}\left(r^{\left(k-1\right)}\right)\;or\;\text{log}\;f(x)=\sum_{k=1}^{K}\text{log}\;g_{k}\left(r^{\left(k-1\right)}\right),$$

*where* $g_{k}=dG_{k}/d\mu$ *is the density of* $G_{k},r^{\left(k-1\right)}=G_{k-1}\circ \cdots \circ G_{1}(x)$, *is the residual for* x *after subtracting $G_{1}⊕\cdots ⊕G_{k-1}$*, *and in particular* $r^{\left(0\right)}=x$. *In other words, the density* f(x) *is exactly the product of the fitted density of each weak learner evaluated at the corresponding sequence of residuals*.

#### 2.3.2 A generative model for F.

It turns out that one can use the classical idea of inverse-CDF sampling to construct a generative model for F as a result of Theorem 2. Specifically, we can generate samples from F by first generating $U\sim \text{Unif}\left((0,1{]}^{d}\right)$ and then computing the following transform

(7) $$F^{-1}\left(U\right)≔G_{1}^{-1}\circ \cdots \circ G_{K}^{-1}\left(U\right),$$

where $G_{k}^{-1}$ is the corresponding inverse for the tree-CDF $G_{k}$ for k=1,2,…,K. To implement the sampler, we next obtain the analytic form of the inverse of a tree-CDF.

Recall that in Section 2.2.1 we showed that a tree-CDF G for a measure $G\in {𝒫}_{T}$ can be expressed as the composition of a sequence of local move mappings $G_{A}:A\to A$ along each subbranch of T. The inverse of the local move mapping $G_{A}$ can be expressed as $G_{A}^{-1}(y)=\left(G_{A,1}^{-1}(y),\ldots ,G_{A,d}^{-1}(y)\right)$ for any $y=\left(y_{1},\ldots ,y_{d}\right)\in A$, where

$$G_{A,j}^{-1}\left(y_{j}\right)=\left\{\begin{array}{ll} y_{j} & \left(j\neq j^{*}\right),\\ G_{A,j}^{\prime -1}\left(\frac{y_{j}-a_{j}}{b_{j}-a_{j}}\right) & \left(j=j^{*}\right), \end{array}\right.$$

and

$$G_{A,j}^{\prime -1}\left(z_{j}\right)=\left\{\begin{array}{ll} a_{j}+\frac{c_{j}-a_{j}}{G\left(A_{l}|A\right)}z_{j} & \text{if}\;y_{j}\leq a_{j}+G\left(A_{l}|A\right)\left(b_{j}-a_{j}\right),\\ c_{j}+\frac{b_{j}-c_{j}}{G\left(A_{r}|A\right)}\left\{z_{j}-G\left(A_{l}|A\right)\right\} & \text{if}\;y_{j}>a_{j}+G\left(A_{l}|A\right)\left(b_{j}-a_{j}\right). \end{array}\right.$$

With the inverse local move function $G_{A}^{-1}$ available for all A∈𝒩(T), we can obtain the explicit form for the inverse tree-CDF $G^{-1}$ as

$$G^{-1}\left(y\right)=G^{\left(R-1\right)}\circ \cdots \circ G^{\left(1\right)}\left(y\right),$$

where for k=1,…,R-1,

$$G^{\left(k\right)}\left(y\right)=\sum_{A\in {𝒜}^{k}}G_{A}^{-1}\left(y\right)1_{A}\left(y\right).$$

### 2.4 Decision-theoretic Considerations

In this subsection we show that our boosting algorithm can be interpreted as fitting the additive model in Equation 6 by sequentially reducing the Kullback-Leibler divergence.

Let $F^{*}$ be the true sampling distribution for the observations and $f^{*}=dF^{*}/d\mu$ its density function. Again, let $F=G_{1}⊕\cdots ⊕G_{K}$ be the additive model for the distribution and f=dF/dμ its density. We consider the entropy loss, i.e., the Kullback-Leibler (KL) divergence between $F^{*}$ and F defined as

(8) $$\text{KL}\left(F^{*}\|F\right)=\int \text{log}\;\frac{f^{*}}{f}dF^{*}.$$

The next lemma states that the entropy loss in Equation 8 can be decomposed into K components (ignoring a constant) each of which only depends on $G_{k}$.

**Lemma 2.** *The Kullback-Leibler divergence can be written as*

(9) $$\text{KL}\left(F^{*}\|F\right)=\int \text{log}\;f^{*}dF^{*}-\sum_{k=1}^{K}\left\{\text{KL}\left({\tilde{F}}_{k}\|\mu \right)-\text{KL}\left({\tilde{F}}_{k}\|G_{k}\right)\right\}.$$

*where* ${\tilde{F}}_{k}$ *is the true distribution of the residualized observation after subtracting* $G_{1},G_{2},\ldots ,G_{k-1}$. *That is*, ${\tilde{F}}_{k}$ *is the true distribution of* $r^{\left(k-1\right)}=G_{k-1}\circ \cdots \circ G_{1}(X)$, *where* $X\sim F^{*}$.

Remark: Note that because $X\sim F^{*}$, we have ${\tilde{F}}_{1}=F^{*}$, and for $k=2,\ldots ,K,{\tilde{F}}_{k}$ is in the following form

$${\tilde{F}}_{k}\left(B\right)=F^{*}\left(G_{1}^{-1}\circ \cdots \circ G_{k-1}^{-1}\left(B\right)\right)\;\text{for all}\;B\in ℬ\left((0,1{]}^{d}\right).$$

The first term on the right-hand side of Equation (9) is a constant. The summand in the second term is positive as long as the measure $G_{k}$ is closer to ${\tilde{F}}_{k}$ than the uniform measure μ in terms of KL divergence. Hence, unless ${\tilde{F}}_{k}=\mu$, the entropy loss could be reduced by adding an additional measure $G_{k}$ that is closer to ${\tilde{F}}_{k}$ than μ. In this way, fitting a measure $G_{k}$ to the residuals $r^{\left(k\right)}$ in the kth step of our boosting algorithm can be understood as an operation to sequentially reduce the KL divergence. Next we turn from the above insight at the population level to the practical strategy at the finite-sample level for fitting F based on n i.i.d. observations ${\left\{x_{i}\right\}}_{i=1}^{n}$ from $F^{*}$. First note that minimizing the divergence $\text{KL}\left(F^{*}\|F\right)$ is equivalent to maximizing the average log-density $\int \;\text{log}\;fdF^{*}$. Thus with a finite sample, we aim to maximize the sample (average) log-density of the training data, that is,

$$\frac{1}{n}\sum_{i=1}^{n}\text{log}\;f\left(x_{i}\right).$$

It follows from Proposition 4 that the log-density can also be decomposed into the sum of K components, which we call “improvements”.

**Lemma 3.** *The sample average log-density can be written as*

$$\frac{1}{n}\sum_{i=1}^{n}\text{log}\;f\left(x_{i}\right)=\sum_{k=1}^{K}D_{k}^{\left(n\right)}\left(G_{k}\right),$$

*where for* k=1,2,…,K, *the improvement $D_{k}^{\left(n\right)}\left(G_{k}\right)$ is*

$$D_{k}^{\left(n\right)}\left(G_{k}\right)=\frac{1}{n}\sum_{i=1}^{n}\text{log}\;g_{k}\left(r_{i}^{\left(k-1\right)}\right)\;\text{with}\;r_{i}^{\left(k-1\right)}=G_{k-1}\circ \cdots \circ G_{1}\left(x_{i}\right).$$

Accordingly, the next proposition characterizes the “optimal” pair $\left(G_{k},T_{k}\right)$ that maximizes $D_{k}^{\left(n\right)}\left(G_{k}\right)$.

**Proposition 5.** *A pair of* $\left(G_{k},T_{k}\right)$ *maximizes* $D_{k}^{\left(n\right)}\left(G_{k}\right)$ *if and only if*

(10) $$T_{k}\in \underset{T\in 𝒯}{\text{arg}\;\text{max}}\sum_{A\in ℒ\left(T\right)}{\tilde{F}}_{k}^{\left(n\right)}\left(A\right)\;\text{log}\;\frac{{\tilde{F}}_{k}^{\left(n\right)}\left(A\right)}{\mu \left(A\right)},$$

*and*

(11) $$G_{k}\left(A\right)={\tilde{F}}_{k}^{\left(n\right)}\left(A\right)\;\text{for all}\;A\in ℒ\left(T_{k}\right),$$

*where* ${\tilde{F}}_{k}^{\left(n\right)}$ *is the empirical measure of the residuals* $r^{\left(k-1\right)}={\left\{r_{i}^{\left(k-1\right)}\right\}}_{i=1}^{n}$. *That is*,

$${\tilde{F}}_{k}^{\left(n\right)}\left(B\right)=\frac{1}{n}\sum_{i=1}^{n}\delta_{B}\left(r_{i}^{\left(k-1\right)}\right)\;\text{for}\;B\in ℬ\left((0,1{]}^{d}\right).$$

Remark 1: The summation in Equation 10 represents the KL divergence between two discrete probability measures with masses given by ${\left\{{\tilde{F}}_{k}^{\left(n\right)}(A)\right\}}_{A\in ℒ(T)}$ and $\{\mu (A){\}}_{A\in ℒ(T)}$ respectively. Equation 10 implies that the “optimal” tree $T_{k}$ should allow maximal differentiation in KL divergence between the induced discretizations of ${\tilde{F}}^{\left(n\right)}$ and μ on its leaves. This proposition offers practical guidance on how to choose a good weak learner, which will be detailed in Section 2.7.1.

Remark 2: As suggested in Lemma 3, the loss is reduced at each step as long as the improvement $D_{k}^{\left(n\right)}\left(G_{k}\right)$ is positive, and the improvement is maximized by the measure described in Proposition 5. However, employing the “optimal” base learner in fitting $G_{k}$ as prescribed in Proposition 5 will generally lead to over-fitting. As in supervised boosting (Hastie et al., 2009), additional regularization is necessary to reduce the variance of the weak learner, and this can be achieved in analogy to supervised boosting through shrinkage toward the “zero”, here the uniform distribution. As will be detailed in Section 2.7.2, one can still ensure the improvement is positive when shrinkage is incorporated in an appropriate way.

### 2.5 Group structure of tree-CDFs

In the previous sections we have defined the new operation for adding two distributions, and discussed the “group-like” structure on probability measures it induces. Here we formalize this notion by showing that the collection of tree-CDFs indeed forms a group.

Proposition 6. *Let* 𝒢 *be a set of tree-CDFs defined as follows*

$$𝒢=\left\{G|G\;\text{is a tree-CDF of}\;G\in {𝒫}_{T}\;\text{for a finite tree}\;T\right\}.$$

*Then*, 𝒢 *generates a group under the composition* ∘. *Specifically, the identity map*—*which corresponds to the uniform distribution—is the identity in the group*. 𝒢 *is closed under* ∘ *and each element has an inverse in* 𝒢.

This group structure is an example of the group-theoretic structure Inouye and Ravikumar (2018) introduced to the family of density destructor transformations, though they did not show in the particular case of tree density destructors the group structure exists. Also note that this group is not abelian, because ∘ is not commutative. This is distinct from the usual group structure defined on the class of tree regressions in supervised settings, as the usual notion of addition is commutative. Boosting based on forward-stepwise fitting of an additive ensemble of elements in 𝒢 does not require the group to be abelian.

### 2.6 Connection to Gradient Boosting

Many of the existing boosting algorithms can be regarded as iterative optimization of loss functions with gradient descent (Mason et al., 1999; Friedman, 2001). In this subsection we discuss our new boosting from this perspective and clarify the difference from existing gradient boosting methods.

First we note that as in gradient boosting, our new algorithm can be seen as fitting a linear additive expansion. As shown in Proposition 4, for the ensemble measure $F=G_{1}⊕\cdots ⊕G_{K}$, the log-density of the ensemble measure f=dF/dμ evaluated at the observation $x_{i}(i=1,\ldots ,n)$ can be decomposed as follows:

$$\text{log}\;f\left(x_{i}\right)=\sum_{k=1}^{K}\text{log}\;g_{k}\left(r_{i}^{\left(k-1\right)}\right),$$

where $g_{k}=dg_{k}/d\mu$ is the density of $G_{k}$ and $r_{i}^{\left(k-1\right)}=G_{k-1}\cdots \circ G_{1}\left(x_{i}\right)$ is the residual. From this expression we can see that log f bears resemblance to a “linear additive model” with which we estimate the log-density function log $f^{*}$ of the unknown measure $F^{*}$. The fitting of this estimate to the data is evaluated with the sum of the log densities

$$L\left(\text{log}\;f\right)=\sum_{i=1}^{n}-\text{log}\;f\left(x_{i}\right).$$

Note that Rosset and Segal (2002) also proposes an additive model for density estimation with the same objective function, but their model is a weighted sum of density functions so is different from our model. We also note that the input of log $g_{k}$ is the residual $r_{i}^{\left(k-1\right)}$ instead of $x_{i}$ itself due to the definition of our new addition rule that involves transformation with the tree-CDFs.

Suppose that we want to update log f, a current estimate of log $f^{*}$, by adding a new density function log g, where g is the density function of the new tree measure G, to improve the fitting. The standard approach in the gradient boosting is evaluating a gradient at the current estimate and approximating its negative with the new function (Mason et al., 1999; Friedman, 2001). In our case, the gradient is constant: for all i,

$${\left[\frac{\partial L}{\partial \;\text{log}\;f(x)}\right]}_{\text{log}\;f(x)=\text{log}\;f\left(x_{i}\right)}=-1.$$

Approximating its negative, 1, with the new function log g is not reasonable. However, this result implies that we can maximize the improvement in the loss function by maximizing the sum of the log-densities evaluated at the current residuals ${\left\{r_{i}^{\left(K-1\right)}\right\}}_{i=1}^{n}$,

$$\sum_{i=1}^{n}\text{log}\;g\left(r_{i}^{\left(K-1\right)}\right),$$

or equivalently their average, which is exactly what our proposed boosting algorithm iteratively does to fit the ensemble measure (see Section 2.4). Therefore, while our new boosting method constructs the ensemble measure in a different way from standard gradient boosting, one can still justify our algorithm as a sequential optimization of the average log densities.

### 2.7 Practical Considerations

In this subsection we describe several practical considerations in implementing and applying the boosting algorithm. While they might first appear as technical details, we have found that they are critical in achieving competitive performance and thus worth elaborating on. Several of these considerations are drawn from similar approaches in supervised boosting.

#### 2.7.1 Choice of a Weak Learner

Searching over all possible trees to solve Equation 10 in each step of the FS algorithm is computationally prohibitive. Nevertheless, Proposition 5 provides hints on how to choose good weak learners that improve the KL divergence efficiently over the iterations. The simplest possible choice of a weak learner, as is often implemented in supervised boosting is to implement a top-down greedy tree learning algorithm that maximizes Equation 10 one split at a time, as is done in fitting classification and regression trees (CART) (Hastie et al., 2009).

In our numerical examples and software, we adopt a weak learner based on a simplified version of an unsupervised (Bayesian) CART model for probability distributions proposed in Awaya and Ma (2024). Fitting this weak learner uses a stochastic one-step look-ahead strategy to choose splitting decisions on each tree node, which generally produces a closer approximation to the “optimal” tree splits than greedy tree algorithms. See Theorem 4.1 in Awaya and Ma (2024) for an asymptotic justification—as the sample size grows, it produces trees that satisfy Proposition 5 with probability increasing to 1. Additional details about the weak learner can be found in Appendix C.

It is worth emphasizing that because we are only building “weak” learners that extract a small fraction of the distributional structure in each iteration, one does not need to be precisely “optimal” in each iteration. More importantly than being “optimal”, the weak learner should facilitate the appropriate shrinkage to avoid overfitting, which we elaborate in the next subsection.

#### 2.7.2 Regularization through Scale-specific Shrinkage

Just as in supervised boosting, simply adopting the solution for Equation 10 (either exact or approximate) as the fit for $G_{k}$ in each iteration will typically lead to overfitting even when the complexity of the tree $T_{k}$ is restricted to be small. In particular, the fitted density tends to have spikes at or near the training points. To avoid such overfitting, it is necessary to regularize or penalize the non-smoothness in the fit for each $G_{k}$. This can be achieved through shrinkage toward “zero”, or the uniform measure μ, thereby discounting the influence of the residuals (or its empirical measure ${\tilde{F}}_{k}^{\left(n\right)}$) on fitting $G_{k}$. In supervised boosting it is typical to introduce a learning rate $c_{0}\in (0,1]$ that controls how much shrinkage toward zero is applied in each iteration. In the current context, this traditional strategy would correspond to setting

$$G_{k}=\left(1-c_{0}\right)\mu +c_{0}{\tilde{F}}_{k}^{\left(n\right)}.$$

We found that in practice one can further improve upon this shrinkage strategy by allowing different levels of shrinkage at different scales. The intuition is that depending on the smoothness of the underlying function, overfitting can be more (or less) likely to happen in learning local details of the distribution and thus one may benefit from enforcing a level of shrinkage that increases (or not) with the depth in the tree $T_{k}$. Following this intuition, we specify a scale-dependent learning rate as follows

$$c\left(A\right)=c_{0}\cdot {\left(1-{\text{log}}_{2}\;\text{vol}\left(A\right)\right)}^{-\gamma },$$

where A is a node in $T_{k},\text{vol}(A)$ is a volume of A. Then the shrinkage toward the uniform can be specified on each node $A\in 𝒩\left(T_{k}\right)$ in terms of the conditional probability on the children of A

(12) $$G_{k}\left(A_{l}|A\right)=\left(1-c\left(A\right)\right)\mu \left(A_{l}|A\right)+c\left(A\right){\tilde{F}}_{k}^{\left(n\right)}\left(A_{l}|A\right)\;\text{for}\;A\in 𝒩\left(T_{k}\right),\;G_{k}\left(\cdot |A\right)=\mu \left(\cdot |A\right)\;\text{for}\;A\in ℒ\left(T_{k}\right),$$

where $A_{l}$ and $A_{r}$ are the children nodes of A in $T_{k},{\tilde{F}}_{k}^{\left(n\right)}\left(A_{l}|A\right)={\tilde{F}}_{k}^{\left(n\right)}\left(A_{l}\right)/{\tilde{F}}_{k}^{\left(n\right)}(A)$ if ${\tilde{F}}_{k}^{\left(n\right)}(A)>0$ and ${\tilde{F}}_{k}^{\left(n\right)}\left(A_{l}|A\right)=\mu \left(A_{l}|A\right)$ otherwise.

The node-specific learning rate c(A) controls how strongly one “pulls” the empirical measure ${\tilde{F}}^{\left(n\right)}$ toward the uniform measure μ at the corresponding scale of A. It is specified with two tuning parameters $c_{0}\in (0,1]$ and γ≥0. The parameter $c_{0}$ controls the global level of shrinkage, and when γ>0 we introduce stronger shrinkage for small nodes, imposing stronger penalty on local spikes. When γ=0, this shrinkage reduces to the standard single learning rate specification described above. In practice, we recommend setting these tuning parameters by cross-validation.

Our next proposition shows that with shrinkage, the sample average log-density is steadily improved in each step of the FS algorithm until the residual distribution becomes the uniform measure.

**Proposition 7.** *For any finite tree* $T_{k}$, *under the definition of* $G_{k}$ *given in* Equation 12, *the improvement satisfies* $D_{k}^{\left(n\right)}\left(G_{k}\right)\geq 0$ *if* c(A)∈(0,1] *for all* $A\in ℒ\left(T_{k}\right)$ *unless* ${\tilde{F}}_{k}^{\left(n\right)}(A)$ *is indistinguishable from the uniform distribution on the tree* $T_{k}$, *that is*, ${\tilde{F}}_{k}^{\left(n\right)}(A)=\mu (A)$ *for all* $A\in ℒ\left(T_{k}\right)$.

#### 2.7.3 Evaluating Variable Importance

As in supervised learning, it is often desirable to evaluate the contribution of each dimension to the approximation of the unknown measure $F^{*}$. Thus we provide a way to quantify variable importance in a conceptually similar manner to what is often used in supervised boosting (see Hastie et al., 2009). We note that Ram and Gray (2011) also introduced a notion of the variable importance in density trees. While their definition is based on improvement in the $L_{2}$ loss, ours is based on the KL divergence, which is consistent with our earlier decision-theoretic discussion.

Specifically, because our boosting algorithm reduces the KL divergence from the unknown measure $F^{*}$, a natural way of quantifying the importance of a variable is adding up the decrease in the KL divergence due to splitting a tree node in the corresponding dimension. Lemma 3 shows that this quantity can be expressed as the sum of the improvements $D_{k}^{\left(n\right)}\left(G_{k}\right)$. In particular, the improvement $D_{k}^{\left(n\right)}\left(G_{k}\right)$ can be further decomposed over the splits of the tree $T_{k}$ as follows

$$D_{k}^{\left(n\right)}\left(G_{k}\right)=\sum_{A\in 𝒩\left(T\right)}{\tilde{F}}_{k}^{\left(n\right)}\left(A\right)\left\{{\tilde{F}}_{k}^{\left(n\right)}\left(A_{l}|A\right)\;\text{log}\frac{G_{k}\left(A_{l}|A\right)}{\mu \left(A_{l}|A\right)}+{\tilde{F}}_{k}^{\left(n\right)}\left(A_{r}|A\right)\;\text{log}\frac{G_{k}\left(A_{r}|A\right)}{\mu \left(A_{r}|A\right)}\right\},$$

where the empirical measure ${\tilde{F}}_{k}^{\left(n\right)}$ is as defined in Proposition 5. Note that the summation inside of the brackets can be written as

$$\text{KL}\left({\tilde{F}}_{k}^{\left(n\right)}\left(A_{l}|A\right)\|\mu \left(A_{l}|A\right)\right)-\text{KL}\left({\tilde{F}}_{k}^{\left(n\right)}\left(A_{l}|A\right)\|G_{k}\left(A_{l}|A\right)\right),$$

where KL(p‖q)=plog(p/q)+(1-p)log[(1-p)/(1-q)], and it quantifies the extent to which splitting A makes $G_{k}$ closer to the distribution of the residuals. Based on the decomposition, a natural definition of the total contribution of dividing in the jth dimension is

$$I_{G_{k},j}=\sum_{A\in {𝒩}_{j}\left(T\right)}{\tilde{F}}_{k}^{\left(n\right)}\left(A\right)\left\{{\tilde{F}}_{k}^{\left(n\right)}\left(A_{l}|A\right)\;\text{log}\frac{G_{k}\left(A_{l}|A\right)}{\mu \left(A_{l}|A\right)}+{\tilde{F}}_{k}^{\left(n\right)}\left(A_{r}|A\right)\;\text{log}\frac{G_{k}\left(A_{r}|A\right)}{\mu \left(A_{r}|A\right)}\right\},$$

where ${𝒩}_{j}(T)$ represents the collection of all nodes in T that are split in the jth dimension. Finally we can define the importance of the jth variable in the additive measure $F=G_{1}⊕\cdots ⊕G_{K}$ by summing over the variable importance across the $G_{k}$’s:

$$I_{j}=\sum_{k=1}^{K}I_{G_{k},j}.$$

#### 2.7.4 Fitting the Margins and the Copula Separately and Addressing Technical Ties

In the density estimation literature, Lu et al. (2013) suggested a two-stage strategy for estimating multivariate densities using tree-based models, which separately fits the marginal distributions and then the dependence (or copula). From our experience, this strategy can often substantially improve the fit of our unsupervised boosting algorithm.

This two-stage strategy is easy to realize in our algorithm. In the first stage, for each of the dimensions, one can adopt weak learners that are constrained to involving tree-CDFs based on partitions along that single dimension. Computing the residuals with tree-CDFs defined on such a tree only removes the marginal distributions from the observations. “Subtracting” all of the marginal distributions from the original observations results in a sample of residuals representing the remaining distribution with uniform marginals (i.e., the corresponding copula). Then in the second stage, the single-dimension constraint on the partition trees is removed, and tree-CDFs are then fitted to the copula. The final fit is simply the sum, in terms of tree-CDF compositions, of all of the marginals and the copula.

A related practical consideration regards tied values in the training data. (Ties in the margins occur much more frequently than ties that occur simultaneously in all margins and thus the issue is particularly relevant during the fitting of the marginal distributions in this two-stage strategy.) When tied values occur, either from the actual data generative mechanism or due to technical reasons such as rounding, the additive tree model itself will only assume that it is due to the actual data generative mechanism and therefore there must be positive probability mass at those tied values, leading to spikes of estimated densities at those values. In practice, if the data generative mechanism is assumed to be continuous and the ties are due to technical reasons such as rounding, one can avoid this issue with a simple preprocessing step for the training data that “smooths out” those spikes by adding small perturbations before fitting the model. We have found a simple strategy to be effective—when there are ties in the training data at the same value x and the adjacent values are $x_{-}$ and $x_{+}\left(x_{-}<x<x_{+}\right)$, we add uniform perturbation to the training data at x on the support $\left(-\left(x-x_{-}\right)/2,\left(x_{+}-x\right)/2\right)$.

#### 2.7.5 Choosing the number of trees

Following similar strategies for supervised tree boosting, we generally set the learning rate $\left(c_{0}\right)$ relatively small—e.g., 0.1 or 0.01—and incorporate a large number of trees (e.g., hundreds to thousands). While our algorithm is generally robust to overspecification in the number of trees, it is still beneficial—for avoiding excessive computation—to adopt an adaptive stopping strategy, which terminates the boosting algorithm when substantive improvement from additional trees is no longer expected. We adopt a simple strategy to achieve this. In each iteration of the algorithm, we use a portion of the data, for example, 90% of the current residuals, to fit the next weak learner, and use the rest of the data to evaluate this tree measure by computing the average log densities. We use this quantity to measure the improvement in the fit. If the average improvement given by, for example, the most recent 50 trees is non-positive, we terminate the algorithm. This adaptive strategy is effective in all of our numerical experiments—it did not noticeably impact predictive performance while substantially reducing computing time.

### 2.8 Expressive Power of Additive Tree Ensembles

One interesting question is what kind of probability measures can be well approximated by the ensemble when relatively simple (e.g., shallow) tree-based weak learners are combined. This is often referred to as the “expressive power” of the model in the machine learning literature, or the “support” of the model in the statistical literature. The expressive power for several normalizing flows have been analyzed (Huang et al., 2018; Jaini et al., 2019; Kong and Chaudhuri, 2020), and the large support property of linear combinations of classification trees has been established in Breiman (2004). We show next that a similar property holds for our unsupervised tree ensemble under the following set of conditions.

**Assumption 1.** *For* k=1,2,…,K, *the pair of the tree* $T_{k}$ *and the measure* $G_{k}$ *that forms a component of the tree ensemble satisfies the following conditions:*

1. *The tree* $T_{k}$ *can be any finite tree formed by the dyadic splitting rule described in* Equation 2 *and* Equation 3. *That is, it incorporates an axis-aligned splitting rule with flexible split points*.
2. Each $T_{k}$ has at least d+1 leaf nodes, where d is the dimension of the sample space.
3. *The measure* $G_{k}$ *can be any conditionally uniform measure on* $T_{k}$, *namely, for every non-terminal node* $A\in 𝒩\left(T_{k}\right)$, *the conditional probability* $G_{k}\left(A_{l}|A\right)$ *can be any value in* (0, 1).

Most notable in the assumption is the second condition, which is in sharp contrast to theories on single tree-based density models in the statistical literature. For instance, a popular tree-based density model, the Pólya tree (PT) model is shown to have the large support under the assumption that a single tree has infinite depth (Lavine, 1992; Ghosal and Van der Vaart, 2017). This is not surprising in the context of additive trees, however, since Proposition 2 of Breiman (2004) for supervised boosting essentially requires the same condition. With additive ensembles, we can combine small trees to express general continuous distributions, as formally stated in the next theorem.

**Theorem 3.** *Let* $F^{*}$ *be a probability measure that has a bounded density function. Then, under Assumption 1, for any* ϵ>0, *there exists a tree ensemble with a finite number of tree measures*, $G_{1}⊕\cdots ⊕G_{K}$, that approximates $F^{*}$ in terms of the KL divergence with this precision, i.e.,

$$\text{KL}\left(F^{*}\|G_{1}⊕\cdots ⊕G_{K}\right)<\epsilon .$$

### 2.9 Connection to Normalizing Flows

Under our definition of additive tree ensembles, the unknown distribution of the observation is modeled as the transformation of the uniform distribution that takes the form

$$T_{1}\circ \cdots \circ T_{K}\left(U\right),\;U\sim \text{Unif}\left((0,1{]}^{d}\right),$$

where $T_{k}=G_{k}^{-1}$. Given this expression, we can find a connection between our new boosting and the normalizing flow (NF) methods, a class of machine learning algorithms used for density estimation. For comprehensive reviews of the NF, see Papamakarios (2019), Papamakarios et al. (2021), and Kobyzev et al. (2020). In NF methods, one approximates the observation’s distribution with the transformation of known distributions such as the uniform and the Gaussian, and the transformation is represented as a composition of multiple functions. From this viewpoint, our boosting method can be considered an NF method in which we use the inverse tree-CDFs $G_{k}^{-1}$ as base transformations.

In NF methods, the estimated log-density is written in the form of the log-determinant of a flow transformation (Papamakarios et al., 2021). In our case, the log-determinant is identical to the sum of the log-densities that appears in our ensemble formulation. To see this, let $F_{K}$ be a composition of tree CDFs $F_{k}=G_{K}\circ \cdots \circ G_{1}$, which corresponds to the ensemble measure written as $F=G_{1}⊕\cdots ⊕G_{K}$ by Theorem 1. We can rewrite the log-determinant using the density function of the tree measures $g_{k}=dG_{k}/d\mu$ as follows

$$\text{log}\;{\text{det}}_{F}(x)=\sum_{k=1}^{K}\text{log}\;{\;\text{det}}_{G_{k}}\left(r^{\left(k-1\right)}\right)=\sum_{k=1}^{K}\text{log}\;g_{k}\left(r^{\left(k-1\right)}\right),$$

where $r^{\left(k-1\right)}=G_{k-1}\circ \cdots \circ G_{1}(x)$. The first equality follows from the chain rule, and the second from the fact that $\left|\text{det}G_{k}(x)\right|=g_{k}(x)$ (almost everywhere). The last expression is the sum of the log-densities in our boosting algorithm (see Proposition 4).

From an algorithmic perspective, some NF methods are similar to our boosting algorithm in that they employ an iterative fitting approach. That is, they sequentially transform the observations to make the distribution closer to the known distributions, as we sequentially residualize the observations with $G_{k}$’s. In particular, Inouye and Ravikumar (2018) introduces an NF method called “density destructors”, which “substracts” information from i.i.d. samples until they become uniform samples. Notably, one particular type of density destructors—the “tree density destructors”—is defined based on subtracting a tree-based transforms, which is equivalent to our concept of tree-CDF transform. The sequential training on the residuals is feasible due to the underlying group structure over the tree-CDF transforms. It substantially reduces the computational cost compared to methods that require stochastic gradient descent for training.

For most NF models, there tends to be a trade-off between the ease in evaluating the fitted density or generating samples from the fitted distribution and that in achieving large expressive power (Papamakarios et al., 2021). It is worth noting that density evaluation and simulation given the fitted additive model are both straightforward to implement under the proposed ensemble model because these tasks only require transforming inputs with the tree-CDF and its inverse function respectively, both of which are available in closed forms. The computational cost of these tasks is O(RK) and in practice, the cost is much smaller because the node splitting is often terminated in shallow levels on much of the sample space. At the same time, the proposed additive tree ensemble is capable of expressing or approximating general continuous distributions as shown in Section 2.8.

## 3. Numerical Experiments

We conduct numerical experiments to demonstrate and evaluate our method. We start with a set of simulations under several representative forms of density functions in a 48-dimensional sample space, through which we examine the impact of the tuning parameters—namely, the learning rate which controls global shrinkage, the scale-specific shrinkage parameter which tunes the shrinkage on tree nodes of different sizes, and the number of trees—as well as that of the two-stage strategy on the performance of the algorithm. In addition, we compare the predictive performance of our method with a state-of-the-art single-tree learner as well as the closely related tree density destructor (Inouye and Ravikumar, 2018). Throughout, the performance of various methods is evaluated by the predictive score, i.e., the average fitted log-density on a testing set.

We then provide a comparative study based on several popular benchmark data sets that pitches our method against several state-of-the-art NF methods including both deep-learning based NFs and the tree density destructor. Finally we demonstrate the computation of variable importance in both artificial data sets and the MNIST handwritten digits data (LeCun et al., 1998).

Unless otherwise noted, we adopt the two-stage strategy and the adaptive stopping discussed in Section 2.7.4 and in Section 2.7.5, respectively, and set the maximum number of trees for the first stage (estimation of the marginal distributions) to 100 per dimension and for the second stage (estimation of the dependence structures) to 5,000 unless otherwise stated. The evaluation of the computational cost is done in a single-core AMD EPYC 7002 (2.50GHz) environment.

### 3.1 Simulation Study in 48D Sample Spaces

**Figure 3:**
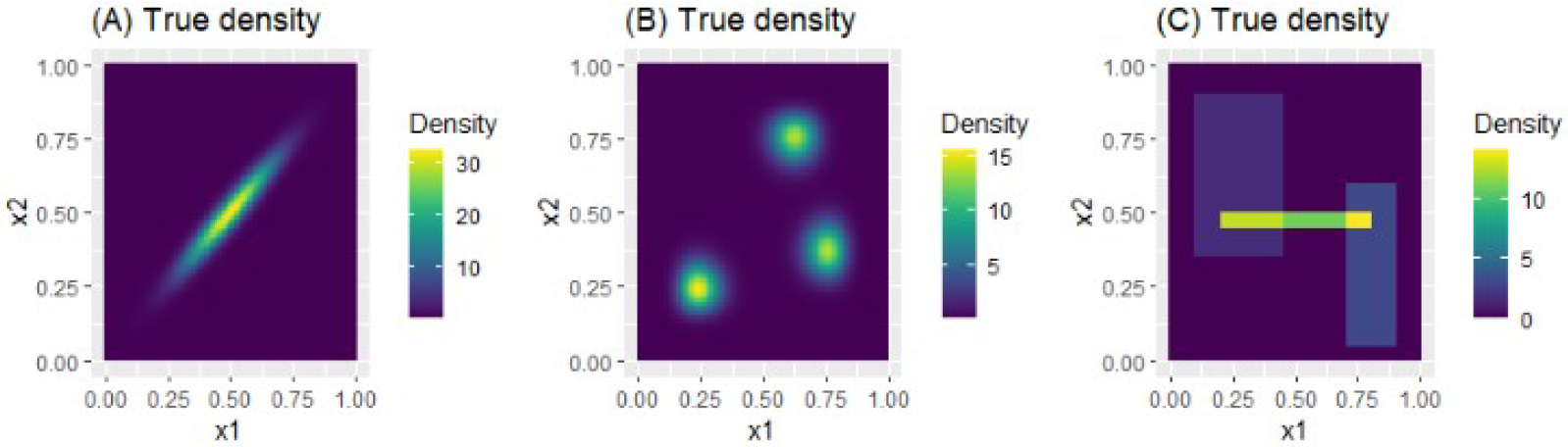
The true marginal densities with respect to $X_{1}$ and $X_{2}$.

We consider three simulation scenarios in a 48-dimensional sample space. The true densities are illustrated in Figure 3. We set the sample size n of both training and testing data sets to 10,000. (Additional details on the data generating process are provided in Appendix D.)

**Figure 4:**
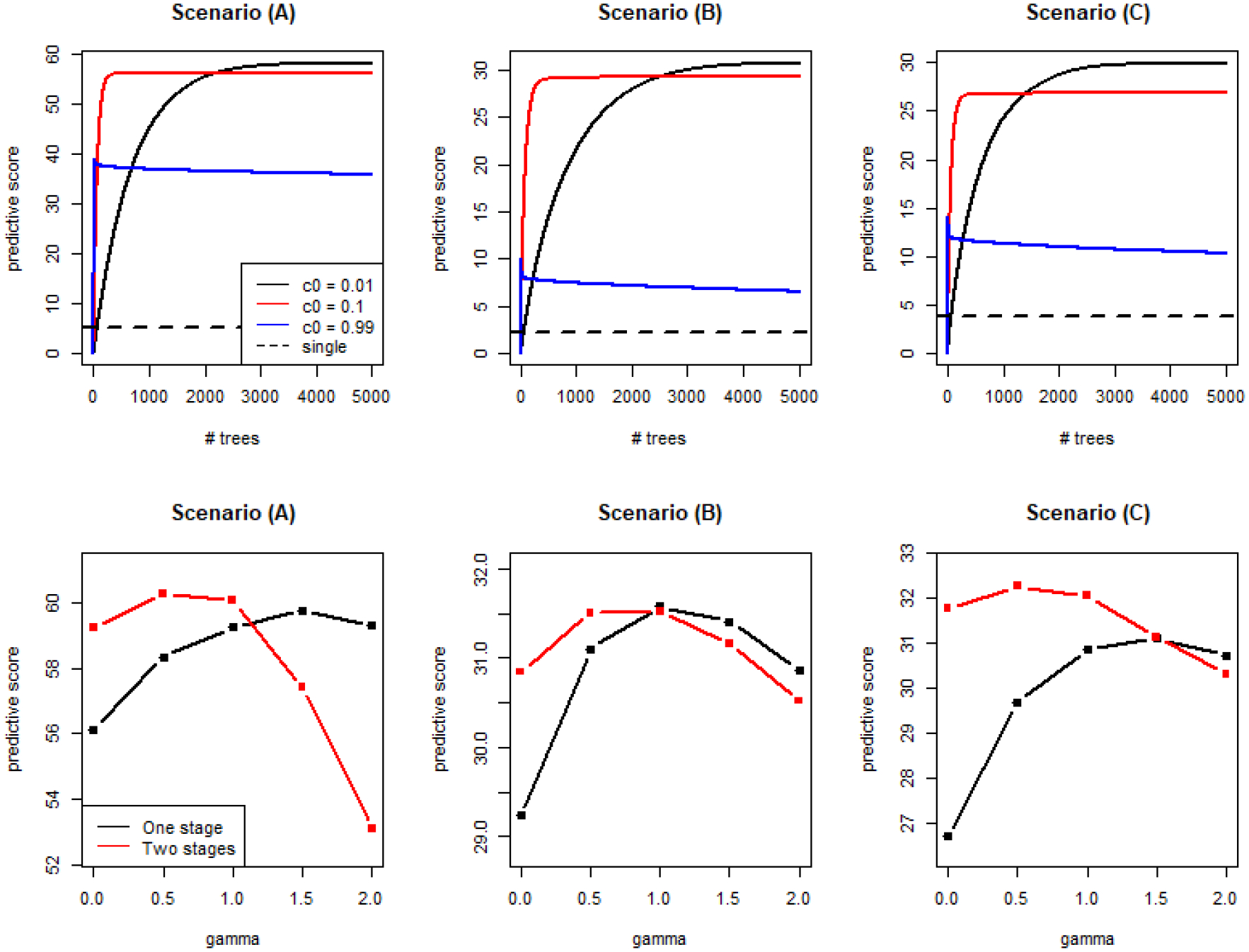
Comparison between the single tree method and the boosting method. The first row presents the predictive performance of the single tree method and the boosting under different $c_{0}$ (global learning rate) and fixed γ=0.0 (scale-specific shrinkage parameter). The second row compares the predictive scores under different γ values when $c_{0}=0.1$. The predictive scores are averages taken under 30 data sets generated with different random seeds.

We evaluate the performance under two types of experimental settings. In the first experiment, we set the value of $c_{0}$, the global shrinkage parameter, to 0.01, 0.1 and 0.99 while setting γ, the scale-specific shrinkage parameter, to 0. Also in this experiment we do not adopt either the two-stage strategy or the adaptive stopping. All these choices are made in order to highlight the effect of changing $c_{0}$ and the number of trees. The predictive performance of the considered methods is visualized in the first row of Figure 4. For completeness, we also report the predictive score for a state-of-the-art single-tree learner (which is essentially a less regularized version of the base learner in our boosting algorithm) to illustrate the dramatic improvement in the performance through fitting an additive ensemble. We can see that our boosting algorithm substantially outperforms the single-tree method in all scenarios, and that the lower learning rate $\left(c_{0}\right)$ generally results in better predictive scores while requiring a larger number of trees. In fact, when $c_{0}$ is small (0.01), the number of trees needed for optimal performance can be as large as 4000 to 5000.

In the second experiment, we compare the performance under different γ (the scale-specific shrinkage parameter) and evaluate the effect of introducing the two-stage strategy. In this experiment γ is set to 0.0, 0.5, …, 2.0, and $c_{0}$ is fixed to 0.1. The results are reported in the second row of Figure 4. Overall one can see that (i) a penalty for small nodes via a positive γ leads to improved fit as long as γ is not too large (i.e., ≥ 1); and (ii) the two-stage strategy can further improve the fit.

**Figure 5:**
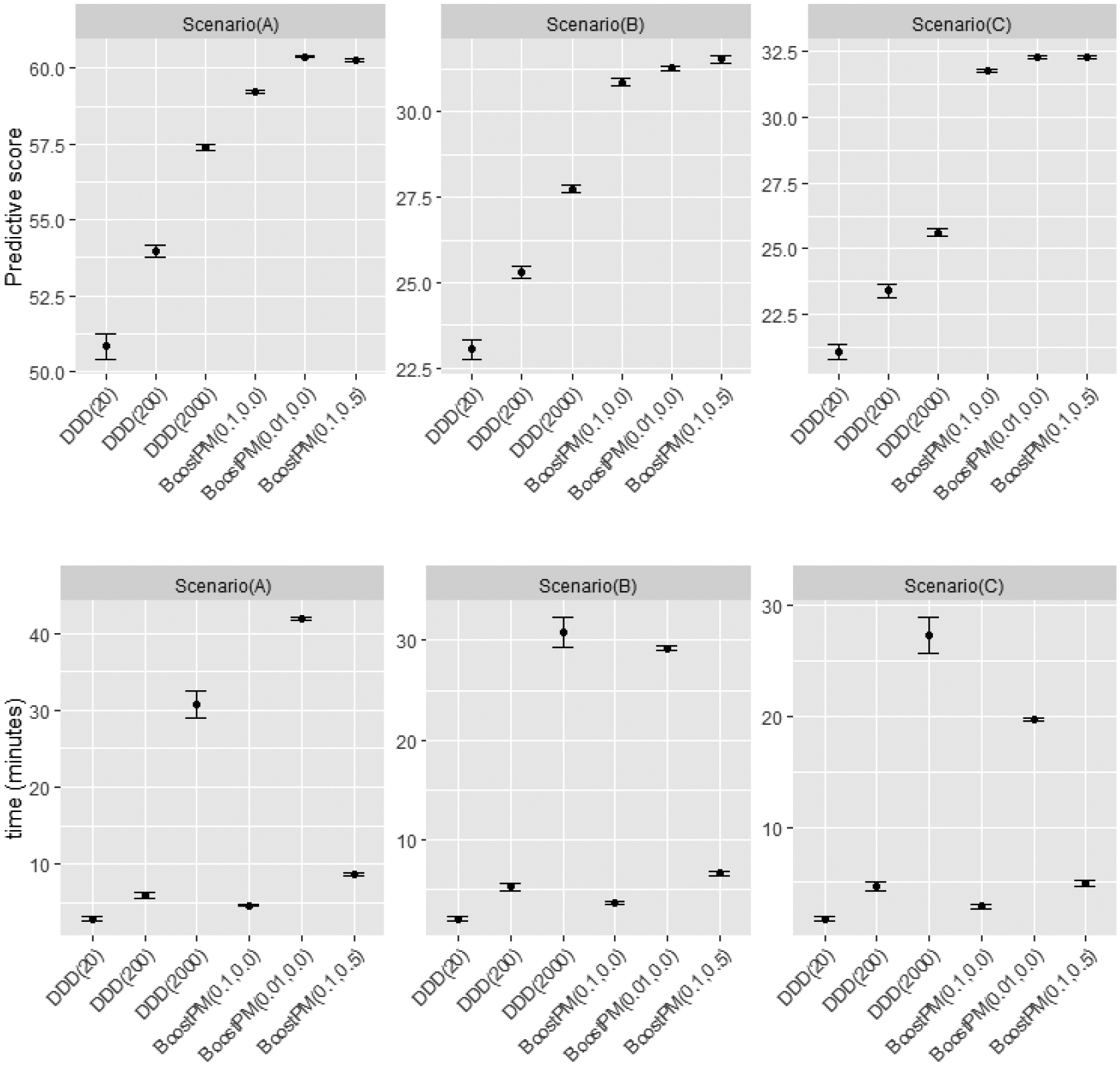
The comparison between the boosting algorithm and the DDD (First row: predictive scores, second row: computation time) based on 30 different data sets. The points correspond to the means, and the intervals are made by adding/subtracting the standard deviations.

In addition, we also provide a comparison with the deep density destructor (DDD) (Inouye and Ravikumar, 2018) using the code available at https://github.com/davidinouye/destructive-deep-learning, in terms of predictive scores and computation cost. For the DDD algorithm, we use the random tree destructor to fit tree measures to the residuals, which is a favorable choice in the numerical experiment provided in Inouye and Ravikumar (2018), and we set the shrinkage parameter (α) to 0.2%, 2%, and 20% of the sample size (20, 200, 2000) following Inouye and Ravikumar (2018). The results are provided in Figure 5. Our algorithm substantially outperforms the DDD and the amount of computational time needed to achieve desirable performance is substantially less under our method. This comparison demonstrates the practical improvement from connecting the tree-based NF approach to boosting and adopting specifications inspired by supervised boosting.

### 3.2 Performance Comparison on Benchmark Data

We evaluate the performance of our boosting algorithm using seven popular benchmark data sets recorded in the University of California, Irvine (UCI) machine learning repository (Dua and Graff, 2017). We preprocessed the four data sets (“POWER”, “GAS”, “HEPMASS”, and “MINIBOONE”) following Papamakarios et al. (2017) with the code provided at https://github.com/gpapamak/maf and the three data sets (“AReM”, “CASP”, and “BANK”) with the code provided at https://zenodo.org/record/4560982#.Yh4k_OiZOCo. We fit the densities and evaluate the predictive scores using the provided code.

We compare our approach with three normalizing flow (NF) methods using deep neural networks to construct transforms, which represent the state-of-the-art for density estimation in machine learning: MADE (Germain et al., 2015), Real NVP (Dinh et al., 2017), MAF (Papamakarios et al., 2017), and DDD (Inouye and Ravikumar, 2018). For MADE, Real NVP and MAF, the predictive scores are referenced from Papamakarios et al. (2017) and Liu et al. (2021), where the detailed settings of the NF models are also provided. For DDD, we evaluate the performance under three possible values of the shrinkage parameter (α), 0.2%, 2%, and 20% of the sample size, and show the best predictive scores. For our boosting algorithm, $\left(c_{0},\gamma \right)$ is set to (0.1, 0.0) and (0.1, 0.5).

A comparison of the predictive scores for our boosting algorithm and the other NF methods is provided in Table 2, where our method is labeled as “boostPM” (which stands for “boosting probability measures”). Our unsupervised tree boosting is overall competitive with the NF methods and even shows the best predictive performance for two data sets (“POWER” and “AReM”). Appendix E also provides a visual comparison of the training data sets and replicated data sets simulated from the fitted generative model, and it confirms that the distributional structures are successfully captured. It should be noted that among the considered methods, ours is the only one that is not based on neural networks but is a combination of the tree-based learners, and therefore requires only a tiny fraction of the computational cost to train. Table 3 presents the computation time on the four large data sets measured in the same single-core environment and shows that our method is substantially faster to train in large n settings. Additional tables are provided in Appendix E which show that the predictive performance of our boosting algorithm is stable under different random seeds (Table 4). In Appendix E, we also present data generated from the fitted tree ensemble following Section 2.3.2 and compare those to the original training data (Figures 8 through 10). The simulated data closely imitates the original training data. Moreover, we report the computation cost for Monte Carlo sampling from the fitted generative model, which is very small compared to the cost of training (Table 5).

### 3.3 Evaluating Variable Importance

Next we demonstrate the use of the variable importance measure introduced in Section 2.7.3. We first carry out an experiment using artificial 10-dimensional distributions of $\left(X_{1},\ldots ,X_{10}\right)$ with sample size n=10,000 under the following scenarios:

**Scenario(1):** $X_{j}\sim \text{Beta}\left(2^{1-j},2^{1-j}\right)$ for j=1,…,10.

**Scenario(2):** Five pairs of random variables $\left(Y_{m,1},Y_{m,2}\right)(m=1,\ldots ,5)$, each of which follows a 2-dimensional Gaussian distribution

$$𝒩\left(\left[\begin{array}{l} 0\\ 0 \end{array}\right],\left[\begin{array}{ll} 1 & \rho \\ \rho & 1 \end{array}\right]\right),$$

with ρ=0.1,0.3,0.5,0.7 and 0.9 respectively, and

$$\left(X_{2(m-1)+1},X_{2m}\right)=\left(\Phi \left(Y_{m,1}\right),\Phi \left(Y_{m,2}\right)\right),$$

where Φ(⋅) is a CDF of the standard Gaussian distribution.

**Scenario(3):** Each of four groups of variables $\left(Y_{1}\right),\left(Y_{2},Y_{3}\right),\left(Y_{4},Y_{5},Y_{6}\right)$, and $\left(Y_{7},Y_{8},Y_{9},Y_{10}\right)$ follows the uni-variate/multi-variate Gaussian distribution in which the marginal distribution is the standard Gaussian and the pairwise correlation is 0.9, and $X_{j}=\Phi \left(Y_{j}\right)$ for j=1,…,10.

**Figure 6:**
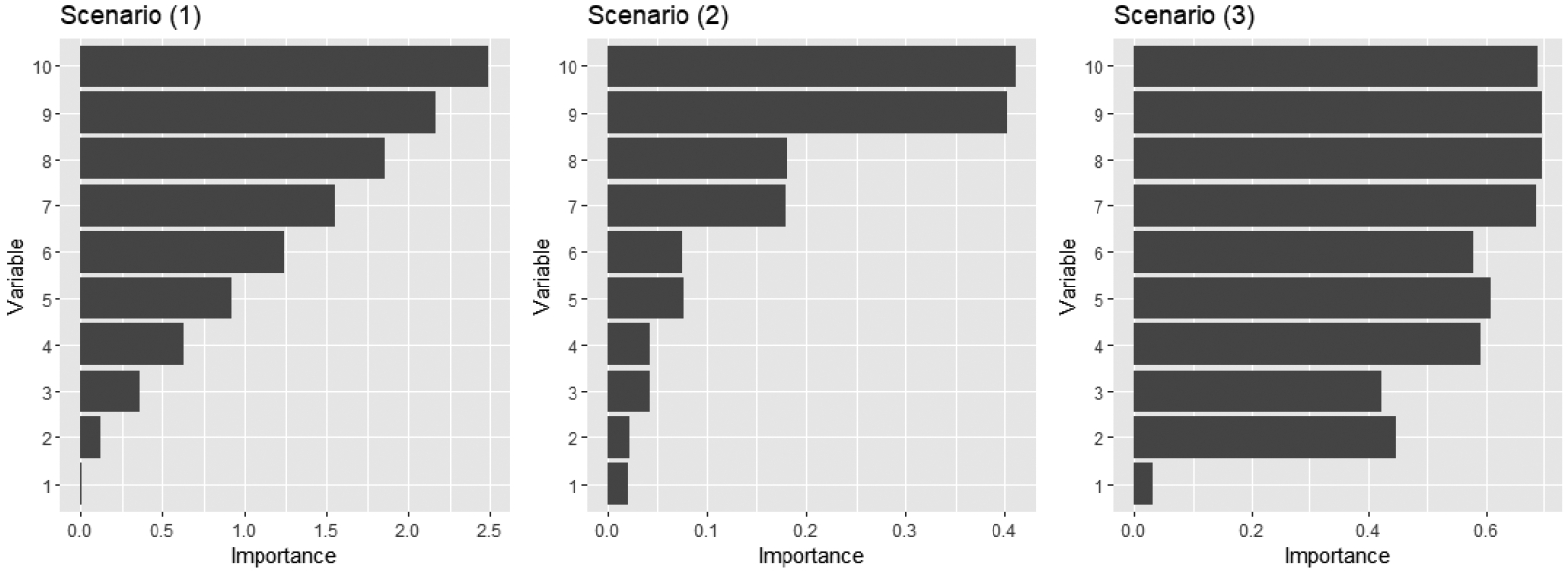
The computed variable importance for the three simulation scenarios.

The computed variable importance is presented in Figure 6. It shows that a variable tends to have higher importance when the marginal distribution is substantially different from the uniform and/or it is strongly correlated with other variables.

We next compute the variable importance on the MNIST handwritten digits data (LeCun et al., 1998). The gray scale images contained in this data consist of 28 × 28 = 784 pixels, and each pixel takes integer values ranging from 0 (black) to 255 (white). We obtain the data with the read_mnist function in the R package dslabs (Irizarry and Gill, 2021) and as in Papamakarios et al. (2017), scale them into [0, 1]. The tuning parameters $c_{0}$ and γ are both set to 0.1.

Recall that our notion of variable importance characterizes how each variable contributes to the deviation from the uniform measure in the underlying sampling distribution. For the particular application of zip-code digit recognition, a pixel is more informative about the underlying digit if it has large variation over the range of intensities. As such, the practical meaning of “importance” in this particular application is the opposite to the statistical importance—it is exactly those pixels with intensities spread out over large ranges (and thus more uniform) that are informative about the underlying digit. As such, we want to emphasize the difference between the “practical importance” and that of the “distributional importance” in terms of KL as we defined before.

**Figure 7:**
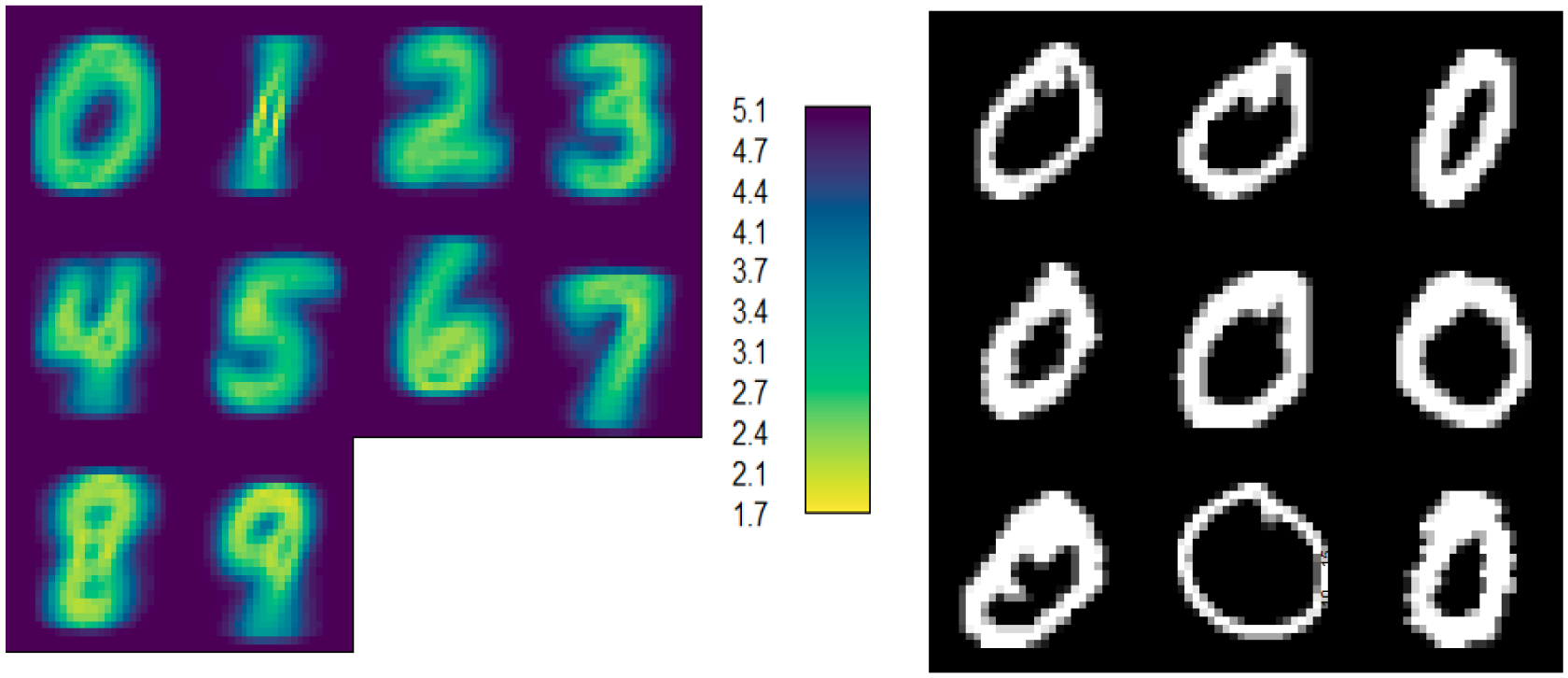
Left: The importance of pixels (variables) computed for the 10 different digits. Yellow/purple colors correspond to low/high importance, which indicates large/small difference in the handwriting styles found at the pixels. Right: A sample of handwriting of 0.

The computed “distributional importance” obtained for the ten different digits is visualized on the left of Figure 7, and a sample of handwriting of 0 is provided on the right. We can see that the pixels with relatively low “distributional importance” and hence high practical importance lie along the outlines of the digits. Hence in this case the “distributional importance” on the left side characterizes “the average shapes” of the handwritten numbers.

## 4. Concluding Remarks

We have proposed an unsupervised boosting method for learning multivariate probability measures by introducing new notions of addition and residuals based on tree-CDF transforms, and demonstrated how one can carry out density estimation and simulate from the fitted measure based on the output of the algorithm. Given its similarity to classical boosting for regression and classification, we expect other techniques for the boosting in such contexts, for example subsampling (Friedman, 2002), could further improve the performance of our boosting method. Due to the limited space, we could not exploit all possible techniques in supervised boosting for improving the performance, but we expect many of them may be effective.

## Supplementary Material

1

## Figures and Tables

**Table 1:** Key concepts in boosting

	Boosting for regression	Boosting for probability measures
Addition	$h_{1}+\cdots +h_{k}$	$G_{1}⊕\cdots ⊕G_{k}$
Residual	$y-\sum_{l=1}^{k-1}h_{l}(x)$	$G_{k-1}\circ \cdots \circ G_{1}(x)$
Zero	0	Uniform distribution on $(0,1{]}^{d}$

**Table 2:** Comparison of the predictive scores (MADE, RealNVP, MAF, DDD, and the proposed algorithm BoostPM with γ=0.0 and 0.5). The means of the estimated log-densities are provided along with the standard errors in parentheses. The two top-performing methods for each data set are indicated in bold font.

	POWER	GAS	HEPMASS	MINIBOONE
MADE	0.40 (0.01)	8.47 (0.02)	−**15.15 (0.02)**	−**12.27 (0.47)**
RealNVP	0.17 (0.01)	8.33 (0.14)	−18.71 (0.02)	−13.55 (0.49)
MAF	0.30 (0.01)	**10.08 (0.02)**	−**17.39 (0.02)**	−**11.68 (0.44)**
DDD	−1.91 (0.03)	−4.40 (0.31)	−28.47 (0.03)	−54.78 (1.03)
BoostPM (0.0)	**1.20 (0.01)**	**9.56 (0.02)**	−20.16 (0.02)	−20.38 (0.46)
BoostPM (0.5)	**1.12 (0.01)**	9.42 (0.02)	−19.43 (0.02)	−17.28 (0.47)
	AReM	CASP	BANK	
MADE	6.00 (0.11)	21.82 (0.23)	14.97 (0.53)	
RealNVP	9.52 (0.18)	**26.81 (0.15)**	26.33 (0.22)	
MAF	9.49 (0.17)	**27.61 (0.13)**	20.09 (0.20)	
DDD	5.73 (0.16)	7.77 (0.19)	9.33 (0.19)	
BoostPM(0.0)	**12.28 (0.16)**	21.84 (0.15)	**36.47 (0.17)**	
BoostPM(0.5)	**12.10 (0.15)**	22.23 (0.15)	**36.34 (0.17)**	

**Table 3:** The training time for a typical run on four benchmark data sets, measured in hours in a single-core AMD EPYC 7002 (2.50GHz) environment. We show the time given under the optimal settings for MADE and RealNVP, and the time under 5 autoregressive layers for MAF. Also provided are the sample size and dimensionality of the data sets.

	POWER	GAS	HEPMASS	MINIBOONE
n	1,659,917	852,174	315,123	29,556
d	6	8	21	43
MADE	3.4	19.3	8.9	0.90
RealNVP	43.7	24.1	60.8	1.4
MAF	15.4	77.5	34.0	0.80
BoostPM(0.0)	0.61	0.75	0.92	0.40
BoostPM(0.5)	2.3	2.1	2.3	1.0

## Data Availability

An R package for our method is available at https://github.com/MaStatLab/boostPM and the code for the numerical examples is at https://github.com/MaStatLab/boostPM_experiments.
