# Supplementary material for: Unsupervised Tree Boosting for Learning Probability Distributions
Source: J Mach Learn Res. Author manuscript; Available in PMC 2026 Jun 10. (PMC13249041)
Supplement: 1 [file NIHMS2165817-supplement-1.pdf]

Appendices

Appendix A. Proofs

In the following proofs, for a tree CDF $\mathbf{G} : (0,1]^d \mapsto (0,1]^d$ and $B \in \mathcal{B}(\Omega)$, the image is denoted by

$$\mathbf{G}(B) = \{\mathbf{G}(x) : x \in B\},$$

and the same notation rule is applied for the inverse $\mathbf{G}^{-1}$. The Lebesgue measure is denoted by μ.

A.1 Proof of Proposition 1

By the assumption on the full support, the CDFs are invertible.

Suppose $X \sim G_1 \oplus \cdots \oplus G_k$. For $x \in (0,1]$, we have

$$\begin{aligned} P(\mathbf{G}_i \circ \cdots \circ \mathbf{G}_1(X) \leq x) &= P(X \leq \mathbf{G}_1^{-1} \circ \cdots \circ \mathbf{G}_i^{-1}(x)) \\ &= \mathbf{G}_k \circ \cdots \circ \mathbf{G}_1(\mathbf{G}_1^{-1} \circ \cdots \circ \mathbf{G}_i^{-1}(x)) \\ &= \mathbf{G}_k \circ \cdots \circ \mathbf{G}_{i+1}(x), \end{aligned}$$

so $\mathbf{G}_i \circ \cdots \circ \mathbf{G}_1(X) \sim G_{i+1} \oplus \cdots \oplus G_k$. The converse can be shown by transforming $P(X < x)$, where $\mathbf{G}_i \circ \cdots \circ \mathbf{G}_1(X)$, in the same way.

□

A.2 Proof of Proposition 2

The tree CDFs are already shown to be bijective in Section 2.2.1, so we only show the measurability here.

Let $\mathcal{E}$ be a set of hyper-rectangles that are written in the form of

$$(a_1, b_1] \times \cdots \times (a_d, b_d]$$

including the null set $\emptyset$. Since $\mathcal{B}((0,1]^d)$ is the Borel σ-field, $E \in \mathcal{B}((0,1]^d)$ holds for every $E \in \mathcal{E}$. To show the measurability of the tree CDF $\mathbf{G}$, which is defined by the measure $G \in \mathcal{P}_T$, it suffices to show that $\mathbf{G}^{-1}(E) \in \mathcal{B}((0,1]^d)$ for every $E \in \mathcal{E}$ since $\mathcal{E}$ generates $\mathcal{B}((0,1]^d)$.

By the definition of $\mathbf{G}$, the image $\mathbf{G}(A)$ ($A \in \mathcal{L}(T)$) is also a hyper-rectangle included in $\mathcal{E}$, and their collection $\{\mathbf{G}(A) : A \in \mathcal{L}(T)\}$ forms a partition of $(0,1]^d$. Hence E is written as a union of disjoint sets:

$$E = \bigcup_{A \in \mathcal{L}(T)} (E \cap \mathbf{G}(A)),$$

where each $E \cap \mathbf{G}(A)$ is a hyper-rectangle that belongs to $\mathcal{E}$ and a subset of $\mathbf{G}(A)$. Hence $\mathbf{G}^{-1}(E \cap \mathbf{G}(A))$ also belongs to $\mathcal{E} \subset \mathcal{B}((0,1]^d)$. Therefore, their finite union

$$\mathbf{G}^{-1}(E) = \bigcup_{A \in \mathcal{L}(T)} \mathbf{G}^{-1}(E \cap \mathbf{G}(A))$$

is also an element of $\mathcal{B}((0,1]^d)$. $\square$

A.3 Proof of Theorem 2

To prove the first assertion, define $\mathbf{G}^{[r]}$ for $r = 1, \ldots, R-1$ as

$$\mathbf{G}^{[r]}(x) = \sum_{A \in \mathcal{A}^r} \mathbf{G}_A(x) \mathbf{1}_A(x).$$

Then the first assertion is equivalent to that if $X \sim G$, then

$$\mathbf{G}^{[1]} \circ \cdots \circ \mathbf{G}^{[R-1]}(X) \sim Unif((0,1]^d), \tag{13}$$

which we prove here. In the following proof, $\mathcal{A}^r$ denotes a collection of nodes that belongs to the rth layer.

In the proof, we let $X^{[R]} = X$ and for $r = 1, \ldots, R-1$

$$X^{[r]} = \mathbf{G}^{[r]} \circ \cdots \circ \mathbf{G}^{[R-1]}(X).$$

Let $G^{[r]}$ denote the distribution of $X^{[r]}$. With these notations, we prove (13) by induction: We show that for A that is a non-terminal node in the rth level, if we have

$$\begin{aligned} &G^{[r+1]}(A_l) = G(A_l),\ G^{[r+1]}(A_r) = G(A_r), \\ &G^{[r+1]}(\cdot \mid A_l) = \mu(\cdot \mid A_l),\ G^{[r+1]}(\cdot \mid A_r) = \mu(\cdot \mid A_r), \end{aligned} \tag{14}$$

then

$$G^{[r]}(A) = G(A),\ G^{[r]}(\cdot \mid A) = \mu(\cdot \mid A). \tag{15}$$

The conditions in Equations (14) holds if $r = R-1$ because $G^{[R]} = G$ and $G \in \mathcal{P}_T$, and the statement in Equation (15) being true for $r = 1$ implies that $G^{[1]} = \mu$, which is equivalent to Equation (13).

Assume Equations (14) holds for some r. By the definition, $\mathbf{G}^{[r]}$ is bijective, and ${\mathbf{G}^{[r]}}^{-1}(A) = A$ for every $A \in \mathcal{A}^r$. Then for $X^{[r]} \sim G^{[r]}$ and $A \in \mathcal{A}^r$, we have,

$$\begin{aligned} G^{[r]}(A) &= P(X^{[r]} \in A) \\ &= P({\mathbf{G}^{[r]}}^{-1}(X^{[r]}) \in {\mathbf{G}^{[r]}}^{-1}(A)) = P(X^{[r+1]} \in A) \\ &= G^{[r+1]}(A_l) + G^{[r+1]}(A_r) = G(A). \end{aligned}$$

Hence the first equation in Equation (15) holds. To prove the second equation, let $X^{[r]} = (X_1^{[r]}, \ldots, X_d^{[r]})$ and

$$A = (a_1, b_1] \times \cdots \times (a_d, b_d].$$

Then, we show that for $z_j \in (a_j, b_j]$,

$$P(X_1^{[r]} \in (a_1, z_1], \ldots, X_d^{[r]} \in (a_d, z_d] \mid X^{[r]} \in A) = \prod_{j=1}^{d} \frac{z_j - a_j}{b_j - a_j} \tag{16}$$

holds. The probability in the left hand side can be written as follows:

$$
\begin{aligned}
&P(X_1^{[r]} \in (a_1, z_1], \dots, X_d^{[r]} \in (a_d, z_d] \mid X^{[r]} \in A) \\
&= P(X_1^{[r]} \in (a_1, z_1], \dots, X_d^{[r]} \in (a_d, z_d] \mid X^{[r+1]} \in A) \\
&= \frac{P(X^{[r+1]} \in A_l)}{P(X^{[r+1]} \in A)} P(X_1^{[r]} \in (a_1, z_1], \dots, X_d^{[r]} \in (a_d, z_d] \mid X^{[r+1]} \in A_l) \\
&\quad + \frac{P(X^{[r+1]} \in A_r)}{P(X^{[r+1]} \in A)} P(X_1^{[r]} \in (a_1, z_1], \dots, X_d^{[r]} \in (a_d, z_d] \mid X^{[r+1]} \in A_r) \\
&= G(A_l \mid A) P(X_1^{[r]} \in (a_1, z_1], \dots, X_d^{[r]} \in (a_d, z_d] \mid X^{[r+1]} \in A_l) \\
&\quad + G(A_r \mid A) P(X_1^{[r]} \in (a_1, z_1], \dots, X_d^{[r]} \in (a_d, z_d] \mid X^{[r+1]} \in A_r). \qquad (17)
\end{aligned}
$$

Let A be divided in the j^*th dimension. By the definition of $\mathbf{G}_A$, for $j \neq j^*$, $X_j^{[r]} \in (a_j, z_j] \iff X_j^{[r+1]} \in (a_j, z_j]$. For j^*, because $\mathbf{G}_{A,j^*}(\cdot)$ is strictly increasing,

$$
\begin{aligned}
X_{j^*}^{[r]} \in (a_{j^*}, z_{j^*}] &\iff \mathbf{G}_{A,j^*}^{-1}\left(X_{j^*}^{[r]}\right) \in \left(\mathbf{G}_{A,j^*}^{-1}(a_{j^*}), \mathbf{G}_{A,j^*}^{-1}(z_{j^*})\right] \\
&\iff X_{j^*}^{[r+1]} \in (a_{j^*}, y_{j^*}],
\end{aligned}
$$

where $y_{j^*} = \mathbf{G}_{A,j^*}^{-1}(z_{j^*})$. The expression of y_{j^*} changes depending on whether $y_{j^*} \leq c_{j^*}$ or not, where c_{j^*} is a partition point at which A is divided. We first assume that $y_{j^*} \leq c_{j^*}$. In this case, the second term in Equation (17) is 0 because $X_{j^*}^{[r+1]} \in (a_{j^*}, y_{j^*}]$ does not happen if $X^{[r+1]} \in A_r$. Also, by the definition of $\mathbf{G}_{A,j^*}$,

$$
\begin{aligned}
\frac{z_{j^*} - a_{j^*}}{y_{j^*} - a_{j^*}} &= \frac{G(A_l \mid A))}{\mu(A_l \mid A)} = G(A_l \mid A))\frac{b_{j^*} - a_{j^*}}{c_{j^*} - a_{j^*}} \\
\iff \frac{y_{j^*} - a_{j^*}}{c_{j^*} - a_{j^*}} &= \frac{1}{G(A_l \mid A))}\frac{z_{j^*} - a_{j^*}}{b_{j^*} - a_{j^*}}.
\end{aligned}
$$

Therefore, it follows that

$$
\begin{aligned}
&P(X_1^{[r]} \in (a_1, z_1], \dots, X_d^{[r]} \in (a_d, z_d] \mid X^{[r]} \in A) \\
&= G(A_r \mid A) \left\{ \prod_{j \neq j^*} \frac{z_j - a_j}{b_j - a_j} \right\} \frac{y_{j^*} - a_{j^*}}{c_{j^*} - a_{j^*}} \\
&= G(A_r \mid A) \left\{ \prod_{j \neq j^*} \frac{z_j - a_j}{b_j - a_j} \right\} \frac{1}{G(A_l \mid A))}\frac{z_{j^*} - a_{j^*}}{b_{j^*} - a_{j^*}} \\
&= \prod_{j=1}^{d} \frac{z_j - a_j}{b_j - a_j}.
\end{aligned}
$$

We can prove (16) for the case of $y_{j^*} > c_{j^*}$ in the same way.

To prove the second result, let $U \sim \text{Unif}((0,1]^d)$. The multi-scale CDF $\mathbf{G}$ is bijective (Proposition 2), so we obtain for $B \in \mathcal{B}(\Omega)$

$$\begin{aligned} P(\mathbf{G}^{-1}(U) \in B) &= P(U \in \mathbf{G}(B)) \\ &= \mu(\mathbf{G}(B)) \\ &= G(B), \end{aligned}$$

where the last line follows Theorem 1. (Note that the proof of Theorem 1 only uses the first result of Theorem 2). Therefore $\mathbf{G}^{-1}(U) \sim G$. □

A.4 Proof of Theorem 1

Let $X \sim G$. By the first result of Theorem 2, we obtain

$$\begin{aligned} G(B) &= P(X \in B) \\ &= P(\mathbf{G}(X) \in \{\mathbf{G}(x) : x \in B\}) \\ &= \mu(\{\mathbf{G}(x) : x \in B\}). \quad \square \end{aligned}$$

A.5 Proof of Lemma 1

We only need to check the countable additivity. By Proposition 2, $\mathbf{G}_k \circ \cdots \circ \mathbf{G}_1$ is bijective. Hence, for disjoint sets $A_l \in \mathcal{B}(\Omega)$ ($l \in \mathbb{N}$), it follows that

$$\mathbf{G}_k \circ \cdots \circ \mathbf{G}_1 \left(\bigcup_l A_l \right) = \bigcup_l \mathbf{G}_k \circ \cdots \circ \mathbf{G}_1 (A_l).$$

Because $\{\mathbf{G}_k \circ \cdots \circ \mathbf{G}_1 (A_l)\}_{l=1,2,\dots}$ are disjoint, this result implies that

$$\begin{aligned} F_k \left(\bigcup_l A_l \right) &= \mu \left(\bigcup_l \mathbf{G}_k \circ \cdots \circ \mathbf{G}_1 (A_l) \right) \\ &= \sum_l \mu(\mathbf{G}_k \circ \cdots \circ \mathbf{G}_1 (A_l)) \\ &= \sum_l F(A_l). \quad \square \end{aligned}$$

A.6 Proof of Proposition 3

First we suppose $X \sim G_1 \oplus \cdots \oplus G_k$. By Proposition 2, $\mathbf{G}_1, \dots, \mathbf{G}_k$ are all bijective, so for $i = 1, \dots, k$, the composition

$$\mathbf{F}_i = \mathbf{G}_i \circ \cdots \circ \mathbf{G}_1$$

is also bijective. For $B \in \mathcal{B}(\Omega)$, we obtain

$$\begin{aligned} P(\mathbf{F}_i(X) \in B) &= P(X \in \mathbf{F}_i^{-1}(B)) \\ &= F_k((\mathbf{F}_i^{-1}(B))) \\ &= \mu(\mathbf{F}_k(\mathbf{F}_i^{-1}(B))) \\ &= \mu(\mathbf{G}_k \circ \cdots \circ \mathbf{G}_{i+1}(B)). \end{aligned}$$

Hence $\mathbf{F}_i(X) \sim G_{i+1} \oplus \cdots \oplus G_k$. Showing the converse is now straightforward. □

A.7 Proof of Proposition 4

We first show the following lemma, which implies that the conditional distributions F_{k-1} and F_k $(k = 2, \ldots, K)$ are the same on subsets in a partition defined by T_k and $\mathbf{F}_{k-1} = \mathbf{G}_{k-1} \circ \cdots \circ \mathbf{G}_1$.

Lemma 4. *Let $\mathcal{L}(T_k)$ be a set of the terminal nodes in T_k. Also, we let $A' \in \mathcal{L}(T_k)$ and $A = \mathbf{F}_{k-1}^{-1}(A')$. Then for any $B \subset A$, $F_{k-1}(B \mid A) = F_k(B \mid A)$.*

(Proof) For $B \subset A$, by Theorem 1, we have

$$\begin{aligned} F_k(B) &= \mu(\mathbf{F}_k(A)) \\ &= \mu(\mathbf{G}_k(\mathbf{F}_{k-1}(B))) \\ &= G_k(\mathbf{F}_{k-1}(B)). \end{aligned} \tag{18}$$

Since $B \subset A$, $\mathbf{F}_{k-1}(B) \subset \mathbf{F}_{k-1}(A) = A'$. Hence $F_k(B)$ is further rewritten as follows

$$\begin{aligned} F_k(B) &= G_k(A' \cap \mathbf{F}_{k-1}(B)) \\ &= G_k(A')G_k(\mathbf{F}_{k-1}(B) \mid A') \\ &= G_k(A')\mu(\mathbf{F}_{k-1}(B) \mid A') \\ &= G_k(A')\frac{\mu(\mathbf{F}_{k-1}(B))}{\mu(A')} \\ &= G_k(A')\frac{F_{k-1}(B)}{\mu(A')}. \end{aligned}$$

By the definition of A, $F_{k-1}(A) = \mu(\mathbf{F}_{k-1}(A)) = \mu(A')$, and by replacing B with A in (18), we have

$$F_k(A) = G_k(\mathbf{F}_{k-1}(A)) = G_k(A').$$

Therefore, we obtain

$$\begin{aligned} F_k(B \mid A) &= \frac{F_k(B)}{F_k(A)} \\ &= G_k(A')\frac{F_{k-1}(B)}{\mu(A')}\frac{1}{F_k(A)} \\ &= \frac{F_{k-1}(B)}{F_{k-1}(A)} \\ &= F_{k-1}(B \mid A). \end{aligned}$$

□

(Proof of Proposition 4) Let $\mathcal{L}_k = \{\mathbf{F}_{k-1}^{-1}(A') : A' \in \mathcal{L}(T_k)\}$. By Lemma 4, the conditional distributions $F_{k-1}(\cdot \mid A)$ and $F_k(\cdot \mid A)$ are the same for $A \in \mathcal{L}_k$. Hence the density functions of F_{k-1} and F_k denoted by f_{k-1} and f_k are expressed as

$$\begin{aligned} f_{k-1}(x) &= \sum_{A \in \mathcal{L}_k} F_{k-1}(A) f_{k-1}(x \mid A)\mathbf{1}_A(x), \\ f_k(x) &= \sum_{A \in \mathcal{L}_k} F_k(A) f_{k-1}(x \mid A)\mathbf{1}_A(x), \end{aligned}$$

where $\mathbf{1}$ is the indicator function. Fix $x \in (0,1]^d$ and let $A_k \in \mathcal{L}_k$ such that $x \in A_k$ and $A'_k = \mathbf{F}_{k-1}(A_k)$. By Theorem 1, we have

$$\begin{aligned} F_{k-1}(A_k) &= \mu(\mathbf{F}_{k-1}(A_k)) = \mu(A'_k), \\ F_k(A_k) &= \mu(\mathbf{F}_k(A_k)) \\ &= \mu(\mathbf{G}_k \circ \mathbf{F}_{k-1}(A_k)) \\ &= G_k(\mathbf{F}_{k-1}(A_k)) \\ &= G_k(A'_k). \end{aligned}$$

Hence, we have

$$\frac{f_k(x)}{f_{k-1}(x)} = \frac{F_k(A_k)}{F_{k-1}(A_k)} = \frac{G_k(A'_k)}{\mu(A'_k)}. \tag{19}$$

Since $x \in A_k$,

$$\mathbf{F}_{k-1}(x) = \mathbf{G}_{k-1} \circ \cdots \circ \mathbf{G}_1(x) \in \mathbf{F}_{k-1}(A_k) = A'_k.$$

Thus the density ratio in (19) is rewritten as

$$\begin{aligned} \frac{f_k(x)}{f_{k-1}(x)} &= G_k(A'_k)\mu(\mathbf{G}_{k-1} \circ \cdots \circ \mathbf{G}_1(x) \mid A'_k) \\ &= G_k(A'_k) g_k(\mathbf{G}_{k-1} \circ \cdots \circ \mathbf{G}_1(x) \mid A'_k) \\ &= g_k(\mathbf{G}_{k-1} \circ \cdots \circ \mathbf{G}_1(x)), \end{aligned}$$

where the second equation follows that $A'_k \in \mathcal{L}(T_k)$. Because the discussion above holds for $k = 2, \ldots, K$, we obtain the following expression

$$\begin{aligned} f_K(x) &= f_1(x) \prod_{k=2}^{K} \frac{f_k(x)}{f_{k-1}(x)} \\ &= f_1(x) \prod_{k=2}^{K} g_k(\mathbf{G}_{k-1} \circ \cdots \circ \mathbf{G}_1(x)). \end{aligned}$$

□

A.8 Proof of Proposition 6

The associativity clearly holds, and showing the existence of the identity element is also straightforward because the identity transformation, which is a tree-CDF of the uniform distribution, is included in $\mathcal{G}$.

Proving the existence of an inverse element for every element in $\mathcal{G}$ is done by showing that an inverse function of a local-move function, which is an essential component of a tree-CDF, is also a local-move function. To this end we use the local-move function $\mathbf{G}_A : A \mapsto A$ defined in Section 2.2.1 and the same notations.

Let $\tilde{c}_{j^*} = a_{j^*} + G(A_l \mid A)(b_{j^*} - a_{j^*})$ and $\tilde{A}_l$ and $\tilde{A}_r$ be a pair of nodes that we obtain by

dividing A at $\tilde{c}_{j^*}$ in the j^*th dimension. Then we define a function $\tilde{\mathbf{G}}_A : A \mapsto A$ such that for any $x \in A$, $\tilde{\mathbf{G}}_A(x) = (\tilde{\mathbf{G}}_{A,1}(x), \ldots, \tilde{\mathbf{G}}_{A,d}(x))$ where $\tilde{\mathbf{G}}_{A,j}(x) = x$ for all $j \neq j^*$, and

$$\frac{\tilde{\mathbf{G}}_{A,j^*}(x) - a_{j^*}}{x_{j^*} - a_{j^*}} = \frac{\mu(A_l|A)}{\mu(\tilde{A}_l|A)} \quad \text{for } x \in \tilde{A}_l \quad \text{and} \quad \frac{b_{j^*} - \tilde{\mathbf{G}}_{A,j^*}(x)}{b_{j^*} - x_{j^*}} = \frac{\mu(A_r|A)}{\mu(\tilde{A}_r|A)} \quad \text{for } x \in \tilde{A}_r.$$

From this definition $\tilde{\mathbf{G}}_A$ works as a local-move function for the conditional distribution that assigns the probability $\mu(A_l|A)$ to $\tilde{A}_l$. It is straightforward to see that this transformation $\tilde{\mathbf{G}}_A$ is identical to $\mathbf{G}_A^{-1}$ (see the expression of the inverse function provided in Section 2.3.2). □

A.9 Proof of Lemma 2

By the definition of the KL divergence, we have

$$\mathrm{KL}(F^*||F) = \int \log f^* dF^* - \int \log f dF^*.$$

By Proposition 4, the second term $\int \log f F^*$ is decomposed as

$$\int \log f dF^* = \sum_{k=1}^{K} \int \log g_k(\mathbf{G}_{k-1} \circ \cdots \circ \mathbf{G}_1(x)) dF^*(x).$$

By the change-of-variable formula (e.g., Theorem 3.6.1 in Bogachev (2007)), the right hand side can be written in a form of integration with respect to $\tilde{F}_k$,

$$\int \log g_k(\mathbf{G}_{k-1} \circ \cdots \circ \mathbf{G}_1(x)) dF^*(x) = \int \log g_k(x) d\tilde{F}_k(x).$$

Since the measure F^* is absolutely continuous with respect to the Lebesgue measure μ, by the definition of $\tilde{F}_k$, that is,

$$\tilde{F}_k(B) = F^*(\mathbf{G}_1^{-1} \circ \cdots \circ \mathbf{G}_{k-1}^{-1}(B)) \quad \text{for all } B \in \mathcal{B}((0,1]^d),$$

$\tilde{F}_k$ is also absolutely continuous with respect to μ. Hence $\tilde{F}_k$ admits the density function denoted by $\tilde{f}_k(x)$, and the right hand side is further rewritten as follows

$$\begin{aligned}\int \log g_k(x) d\tilde{F}_k(x) &= \int \log \frac{\tilde{f}_k(x)}{\mu(x)} d\tilde{F}_k(x) - \int \log \frac{\tilde{f}_k(x)}{g_k(x)} d\tilde{F}_k(x) \\ &= \mathrm{KL}(\tilde{F}_k||\mu) - \mathrm{KL}(\tilde{F}_k||G_k). \quad \square\end{aligned}$$

A.10 Proof of Lemma 3

The result immediately follows Proposition 4, which shows that the log-density of the ensemble measure can be decomposed into a sum of log-densities of the tree-based measures the ensemble consists of.

A.11 Proof of Proposition 5

Since T_k is a finite tree, the log of g_k, which is piece-wise constant on T_K, is written as

$$\log g_k(x) = \sum_{A \in \mathcal{L}(T_k)} \log \frac{G_k(A)}{\mu(A)} \mathbf{1}_A(x) \quad \text{for } x \in (0,1]^d.$$

Hence the improvement $D_k^{(n)}(G_k)$ is rewritten as follows:

$$\begin{aligned} D_k^{(n)}(G_k) &= \sum_{A \in \mathcal{L}(T_k)} \tilde{F}_k^{(n)}(A) \log \frac{G_k(A)}{\mu(A)} \\ &= \sum_{A \in \mathcal{L}(T_k)} \tilde{F}_k^{(n)}(A) \log \frac{\tilde{F}_k^{(n)}(A)}{\mu(A)} - \sum_{A \in \mathcal{L}(T_k)} \tilde{F}_k^{(n)}(A) \log \frac{\tilde{F}_k^{(n)}(A)}{G_k(A)}. \end{aligned}$$

Because the second term in the bottom line takes a form of KL divergence defined for the two discrete distributions, it is minimized if $G_k(A) = \tilde{F}_k^{(n)}(A)$ for all $A \in \mathcal{L}(T_k)$. Under this G_k, since the second term is 0, the improvement is maximized if T_k satisfies the condition provided in Proposition 5. □

A.12 Proof of Proposition 7

In this proof, we suppose the learning rate $c(A)$ is independent to a node A for simplicity. For every leaf node $A \in \mathcal{L}(T_k)$, there is a sequence of nodes $\{B_{A,r}\}_{r=1}^R$ such that $B_{A,r}$ belongs to the rth level of T_k, and

$$(0,1]^d = B_{A,1} \supset B_{A,2} \supset \cdots \supset B_{A,r} = A.$$

With such sequences, based on the discussion in Appendix A.11 , the improvement $D_k^{(n)}(G_k)$ is decomposed as

$$\begin{aligned} D_k^{(n)}(G_k) &= \sum_{A \in \mathcal{L}(T_k)} \tilde{F}_k^{(n)}(A) \log \frac{G_k(A)}{\mu(A)} \\ &= \sum_{A \in \mathcal{L}(T_k)} \tilde{F}_k^{(n)}(A) \left[\log \frac{G_k(B_{A,2}|B_{A,1})}{\mu(B_{A,2}|B_{A,1})} + \cdots + \log \frac{G_k(B_{A,R}|B_{A,R-1})}{\mu(B_{A,R}|B_{A,R-1})} \right] \\ &= \sum_{A \in \mathcal{N}(T_k)} \left[\tilde{F}_k^{(n)}(A_l) \log \frac{G_k(A_l \mid A)}{\mu(A_l \mid A)} + \tilde{F}_k^{(n)}(A_r) \log \frac{G_k(A_r \mid A)}{\mu(A_r \mid A)} \right]. \end{aligned}$$

For the bottom line the summand is 0 if $\tilde{F}_k^{(n)}(A) = 0$. Otherwise, the conditional probabilities $\tilde{F}_k^{(n)}(A_l \mid A)$ and $\tilde{F}_k^{(n)}(A_r \mid A)$ are defined. In such a case, by the definition of

$G_k(A_l \mid A)$,

$$\log \frac{G_k(A_l \mid A)}{\mu(A_l \mid A)} = \log \left[\frac{(1-c)\mu(A_l \mid A) + c\tilde{F}_k^{(n)}(A_l \mid A)}{\mu(A_l \mid A)} \right]$$
$$\geq (1-c)\log 1 + c \log \frac{\tilde{F}_k^{(n)}(A_l \mid A)}{\mu(A_l \mid A)}$$
$$= c \log \frac{\tilde{F}_k^{(n)}(A_l \mid A)}{\mu(A_l \mid A)},$$

where the second line follows the Jensen's inequality. The same result holds for A_r. Hence,

$$\tilde{F}_k^{(n)}(A_l) \log \frac{G_k(A_l \mid A)}{\mu(A_l \mid A)} + \tilde{F}_k^{(n)}(A_r) \log \frac{G_k(A_r \mid A)}{\mu(A_r \mid A)}$$
$$\geq c\tilde{F}_k^{(n)}(A) \left[\tilde{F}_k^{(n)}(A_l \mid A) \log \frac{\tilde{F}_k^{(n)}(A_l \mid A)}{\mu(A_l \mid A)} + \tilde{F}_k^{(n)}(A_r \mid A) \log \frac{\tilde{F}_k^{(n)}(A_r \mid A)}{\mu(A_r \mid A)} \right],$$

where the sum inside of the brackets is the KL divergence for the two Bernoulli distributions and thus non-negative. Therefore, the improvement $D_k^{(n)}(G_k)$ is non-negative. Additionally, the last inequality is strict if and only if $\tilde{F}_k^{(n)}(A_l \mid A) = \mu(A_r \mid A)$ and so $D_k^{(n)}(G_k)$ is positive. □

Appendix B. Expressive Power of the Tree Ensemble

In this section, we provide theoretical results on the expressive power of the tree ensemble with the final goal of proving Theorem 3.

B.1 Preparations

We introduce the following notations:

1. Let $\mathcal{T}^L$ be a collection of dyadic trees with axis-aligned boundaries with at most L maximum resolution. When $L = d$, $\mathcal{T}^d$ is a set of trees that can be formed under Assumption 1. We note that as implied in the following proofs, $\mathcal{T}^L$ can a set of trees that have at least one node reach the Lth while the other leaf nodes belong to the shallower levels.

2. For a tree $T \in \mathcal{T}^L$, a set $\mathcal{P}_T$ denotes a collection of probability measures conditionally uniform on T such that
$$G(\cdot \mid A) = \mu(\cdot \mid A) \text{ and } G(A) > 0 \tag{20}$$
for every terminal node $A \in T$. A collection of such tree measures are denoted by $\mathcal{G}_0^L$, that is,
$$\mathcal{G}_0^L = \{G : G \in \mathcal{P}_T \text{ for some } T \in \mathcal{T}^L\}.$$
For a measure $G \in \mathcal{G}_0^L$ defined on a tree $T \in \mathcal{T}^L$, we can define a tree-CDF as in Section 2, which is denoted by $\mathbf{G}$. We define a set $\boldsymbol{\mathcal{G}}_0^L$ as a collection of such tree CDFs, namely,
$$\boldsymbol{\mathcal{G}}_0^L = \{\mathbf{G} : \mathbf{G} \text{ is a tree CDF of } G \in \mathcal{G}_0^L\}.$$

3. Let $\boldsymbol{\mathcal{G}}^L$ denote a set of finite composition of tree CDFs, that is,

$$\boldsymbol{\mathcal{G}}^L = \{\mathbf{G}_K \circ \cdots \circ \mathbf{G}_1 : K \in \mathbb{N} \text{ and for } k = 1, \ldots, K,\ \mathbf{G}_k \in \boldsymbol{\mathcal{G}}_0^L\},$$

and define $\mathcal{G}^L$ as a collection of probability measures defined by such finite compositions, that is,

$$\mathcal{G}^L = \{\mu(\mathbf{G}(\cdot)) : \mathbf{G} \in \boldsymbol{\mathcal{G}}^L\}.$$

Hence $\mathcal{G}^L$ includes all measures that can be expressed in the form of ensemble $G_1 \oplus \cdots \oplus G_K$.

We also need to review the definition of push-forward measures because this notation is closely related to the operation of residualization. Let φ be a mapping $\Omega \mapsto \Omega$ and H be a probability measure. Then the push-forward of H is defined in the following form:

$$\varphi \# H(B) = H(\varphi^{-1}(B)) \text{ for } B \in \mathcal{B}(\Omega).$$

The following lemma establishes a connection between the ensemble measure and the push-forward measures.

Lemma 5. *For a probability measure F, $F \in \mathcal{G}^L$ holds if and only if there exists a mapping $\mathbf{G} \in \boldsymbol{\mathcal{G}}^L$ such that $\mathbf{G} \# F = \mu$.*

(Proof) Suppose $F \in \mathcal{G}^L$. Then there exists a mapping $\mathbf{G} \in \boldsymbol{\mathcal{G}}^L$ such that

$$F(B) = \mu(\mathbf{G}(B)) \text{ for } B \in \mathcal{B}(\Omega).$$

From Proposition 2, $\mathbf{G}$ is bijective. Hence for $B \in \mathcal{B}(\Omega)$, we have

$$\mu(B) = \mu(\mathbf{G} \circ \mathbf{G}^{-1}(B)) = F(\mathbf{G}^{-1}(B)),$$

so $\mathbf{G} \# F = \mu$. The necessity can be shown in the same way. □

In the rest of the section, we first discuss the expressive power of the tree ensemble for the uni-variate cases and next generalize the result for the multi-variate cases. After that, this result is used to prove Theorem 3.

B.2 Uni-variate Cases

The following proposition shows that any distribution with piece-wise constant and positive densities can be represented in the form of tree ensemble.

Proposition 8. *Let F be a probability measure that admits the piece-wise constant density f with the following form*

$$f(x) = \sum_{i=1}^{I} \beta_i 1_{(c_{i-1}, c_i]},$$

where $\beta_i > 0$ for $i = 1, \ldots, I$ and

$$0 = c_0 < c_1 < \cdots < c_I = 1.$$

Then, if $L \geq 2$, $F \in \mathcal{G}^L$ holds.

(Proof) We first show the existence of a tree CDF $\mathbf{G}_1 \in \boldsymbol{\mathcal{G}}_0^2$ such that the push-forward measure $\mathbf{G}_1\#F$ has a density f_1 with the following form

$$f_1(x) = \sum_{i=1}^{I-1} \tilde{\beta}_i 1_{(\tilde{c}_{i-1}, \tilde{c}_i]}, \tag{21}$$

where $\tilde{\beta}_i > 0$ for $i = 1, \ldots, I-1$ and $0 = \tilde{c}_0 < \tilde{c}_1 < \cdots < \tilde{c}_{I-1} = 1$.

Let $\alpha \in (0,1)$ be a constant that satisfies

$$\frac{1-\alpha}{\alpha} = \frac{\beta_2}{\beta_1}\frac{1-c_1}{c_1}.$$

Then define a measure $G_1 \in \mathcal{G}_0^2$ such that

$$G_1((0, c_1]) = \alpha,\ G_1((c_1, 1]) = 1 - \alpha$$

and G_1 is conditionally uniform on $(0, c_1]$ and $(c_1, 1]$. Let $\mathbf{G}_1$ be G_1's tree CDF and $F_1 = \mathbf{G}_1\#F$ be a probability measure with the density f_1. For $x \in (0, \alpha]$, we have

$$F_1((0,x]) = F(\mathbf{G}_1^{-1}((0,x])) = F((0, \mathbf{G}_1^{-1}(x)]) = \int_0^{\mathbf{G}_1^{-1}(x)} f d\mu.$$

Hence, by the chain rule, the density at this x is written as

$$f_1(x) = \frac{c_1}{\alpha} f\left(\mathbf{G}_1^{-1}(x)\right) = \frac{c_1}{\alpha}\beta_1.$$

Similarly, the density at $x \in (\alpha, 1]$ is written as

$$f_1(x) = \frac{1-c_1}{1-\alpha} f\left(\mathbf{G}_1^{-1}(x)\right).$$

Let $\tilde{c}_i = \mathbf{G}_1(c_{i+1})$ for $i = 1, \ldots, I-1$. By this definition, $\alpha < \tilde{c}_1$, and the density of f_1 at $x \in (\alpha, \tilde{c}_1]$ satisfies

$$f_1(x) = \frac{1-c_1}{1-\alpha}\beta_2 = \frac{c_1}{\alpha}\beta_1,$$

where the second equation follows the definition of α. Hence f_1 is constant on $(0, \tilde{c}_1]$. Moreover, the density on $(\tilde{c}_{i-1}, \tilde{c}_i]$ for $i = 2, \ldots, I-1$ is $(1-c_1)/(1-\alpha)\beta_{i-1}$ so constant. Therefore the density f_1 is written in the form of Equation 21.

By using the same logic for the rest of the $I-2$ discontinuous points, we can define tree CDFs $\mathbf{G}_2, \cdots, \mathbf{G}_{I-1}$ that connect the densities at these points one by one. Hence the measure $(\mathbf{G}_{I-1} \circ \cdots \circ \mathbf{G}_1)\#F$ has a constant density and thus is the uniform measure μ. □

B.3 Multi-variate Cases

In this section, we prove the following proposition that is a generalization of Proposition 8.

Proposition 9. *For $j = 1, \dots, d$, let $\{c_{j,i_j}\}_{i_j=1}^{I_j}$ be a sequence such that*

$$0 = c_{j,0} < c_{j,1} < \cdots < c_{j,I_j} = 1,$$

and $\mathcal{L} = \{A_{i_1,\dots,i_d}\}_{i_1,\dots,i_d}$ be a partition of the sample space $(0,1]^d$ that consists of rectangles written as

$$A_{i_1,\dots,i_d} = (c_{1,i_1-1}, c_{1,i_1}] \times \cdots \times (c_{d,i_d-1}, c_{d,i_d}].$$

If a probability measure F is piecewise uniform on $\mathcal{L}$ and written as

$$F(B) = \sum_{i_1,\dots,i_j} a_{i_1,\dots,i_d} \frac{\mu(B \cap A_{i_1,\dots,i_d})}{\mu(A_{i_1,\dots,i_d})}, \text{ for } B \in \mathcal{B}((0,1]^d),$$

where $a_{i_1,\dots,i_d} > 0$, then for $L \geq d+1$, there is a mapping $\mathbf{G} \in \boldsymbol{\mathcal{G}}^L$ such that $\mathbf{G}\#F = \mu$ and thus $F \in \mathcal{G}^L$. In addition, we can choose $\mathbf{G}$ so that for every pair of indices $(i_1, \dots, i_d)$, the image $\mathbf{G}(A_{i_1,\dots,i_d})$ is a rectangle written as

$$(\mathbf{G}(c_{1,i_1-1}), \mathbf{G}(c_{1,i_1})] \times \cdots \times (\mathbf{G}(c_{d,i_d-1}), \mathbf{G}(c_{d,i_d})].$$

(Proof) We use induction: We assume that the statement of Proposition 9 is valid for the 1,2,...,(d-1)-dimensional cases. Because in this proof we handle measures and transformation defined in different dimensional spaces, the sets $\mathcal{G}^L$ and $\boldsymbol{\mathcal{G}}^L$ defined for the j-dimensional space are denoted by $\mathcal{G}^{L,d}$ and $\boldsymbol{\mathcal{G}}^{L,d}$, respectively.

Inside of the induction, we also assume that for some $l \in \{1, \dots, I_d - 1\}$, there are mappings $\mathbf{G}_1, \dots, \mathbf{G}_l \in \boldsymbol{\mathcal{G}}^{L,d}$ such that a probability measure $F_l := (\mathbf{G}_l \circ \cdots \circ \mathbf{G}_1)\#F$ is a piecewise uniform probability measure written as, for $B \in \mathcal{B}((0,1]^d)$,

$$F_l(B) = \sum_{i=1}^{l} C_i \frac{\mu(B \cap (0,1]^{d-1} \times (c_{d,i-1}, c_{d,i}])}{\mu((0,1]^{d-1} \times (c_{d,i-1}, c_{d,i}])} + \sum_{i_d=l+1}^{I_d} \sum_{i_1,\dots,i_{d-1}} a^{(l)}_{i_1,\dots,i_d} \frac{\mu(B \cap A^{(l)}_{i_1,\dots,i_d})}{\mu(A^{(l)}_{i_1,\dots,i_d})},$$

where $C_i > 0$ and $a^{(l)}_{i_1,\dots,i_d} > 0$ for all indices. Also, for the second term, $A^{(l)}_{i_1,\dots,i_d}$ is a rectangular written as

$$A^{(l)}_{i_1,\dots,i_d} = \left(c^{(l)}_{1,i_1-1}, c^{(l)}_{1,i_1}\right] \times \cdots \times \left(c^{(l)}_{d-1,i_{d-1}-1}, c^{(l)}_{d-1,i_{d-1}}\right] \\ \times (c_{d,I_d-1}, c_{d,I_d}],$$

where for $j = 1, \dots, d-1$, $\{c^{(l)}_{j,i}\}_{i=1}^{I_j}$ is a sequence such that

$$0 = c^{(l)}_{j,1} < c^{(l)}_{j,2} < \cdots < c^{(l)}_{j,I_j} = 1.$$

(We note that this sequence's length can be different from "I_j" provided in Proposition 9 but to avoid an excessive number of indices, we use I_j here because its size does not affect the logic provided in this proof.) Under this assumption, we show that there is a measure ("F_{l+1}") that has the same form for $l+1$.

Define a $d-1$-dimensional probability measure $\hat{F}_{l+1}$

$$\hat{F}_{l+1} = \sum_{i_1,\dots,i_d} \frac{a^{(l)}_{i_1,\dots,i_{d-1},l+1}}{C_{l+1}} \frac{\mu_{d-1}(B \cap \hat{A}^{(l)}_{i_1,\dots,i_{d-1}})}{\mu_{d-1}(\hat{A}^{(l)}_{i_1,\dots,i_{d-1}})} \text{ for } B \in \mathcal{B}((0,1]^{d-1}),$$

where C_{l+1} is the normalizing constant, μ_{d-1} is the Lebesgue measure defined for the $d-1$-dimensional sample space, and $\hat{A}^{(l)}_{i_1,\dots,i_{d-1}}$ is a set written as

$$\hat{A}^{(l)}_{i_1,\dots,i_{d-1}} = \left(c^{(l)}_{1,i_1-1}, c^{(l)}_{1,i_1}\right] \times \cdots \times \left(c^{(l)}_{d-1,i_{d-1}-1}, c^{(l)}_{d-1,i_{d-1}}\right]. \tag{22}$$

Because $\hat{F}$ is a piecewise uniform measure defined on the partition that consists of hyper-rectangles, by the assumption we set for the induction, there is a mapping $\hat{\mathbf{G}}_{l+1} \in \boldsymbol{\mathcal{G}}^{L-1,d-1}$ such that $\hat{\mathbf{G}}_{l+1}\#\hat{F}_{l+1} = \mu_{d-1}$. With this mapping, we define a mapping $\mathbf{G}_{l+1} : (0,1]^d \mapsto (0,1]^d$ such that for $x = (x_1,\dots,x_d) \in (0,1]^d$,

$$\mathbf{G}_{l+1}(x) = \left(\hat{\mathbf{G}}_{l+1}(x_1,\dots,x_{d-1}), x_d\right)$$

if $x_d \in (c_{d,l}, 1]$ and otherwise $\mathbf{G}_{l+1}(x) = x$. The mapping $\mathbf{G}_{l+1}$ moves points only in $(0,1]^{d-1} \times (c_{i,l}, 1]$, which is a node one can obtain by dividing the sample space only once, according to $\hat{\mathbf{G}}_{l+1}$, which is a mapping that is a composition of tree CDFs based on trees with $L-1$ leaf nodes. Hence $\mathbf{G}_{l+1}$ is a composition of tree CDFs defined on trees with $(L-1)+1 = L$ leaf nodes, so we have $\mathbf{G}_{l+1} \in \boldsymbol{\mathcal{G}}^{L,d}$. With this mapping, we define a measure $F_{l+1} = \mathbf{G}_{l+1}\#F_l$.

Fix a pair of indices $(i_1,\dots,i_d)$ and let $B_{d-1} \in \mathcal{B}((0,1]^{d-1})$ and $B_1 \in \mathcal{B}((0,1])$ be measurable sets such that

$$B_{d-1} \times B_1 \in \hat{\mathbf{G}}_{l+1}(\hat{A}^{(l)}_{i_1,\dots,i_{d-1}}) \times (c_{d,i_d-1}, c_{d,i_d}].$$

If $i_d \le l$, by the definition of F_{l+1} and $\mathbf{G}_{l+1}$,

$$F_{l+1}(B_{d-1} \times B_1) = F_l(\mathbf{G}^{-1}_{l+1}(B_{d-1} \times B_1)) = F_l(B_{d-1} \times B_1).$$

On the other hand, if $i_d \ge l+1$, since F_l is conditionally uniform on $A^{(l)}_{i_1,\dots,i_d}$,

$$\begin{aligned} F_{l+1}(B_{d-1} \times B_1) &= F_l(\mathbf{G}^{-1}_{l+1}(B_{d-1} \times B_1)) = F_l(\hat{\mathbf{G}}^{-1}_{l+1}(B_{d-1}) \times B_1) \\ &= a^{(l)}_{i_1,\dots,i_d} \frac{\mu(\hat{\mathbf{G}}^{-1}_{l+1}(B_{d-1}) \times B_1)}{\mu(A^{(l)}_{i_1,\dots,i_d})} \\ &= a^{(l)}_{i_1,\dots,i_d} \frac{\mu_{d-1}(\hat{\mathbf{G}}^{-1}_{l+1}(B_{d-1}))\mu_1(B_1)}{\mu_{d-1}(\hat{A}^{(l)}_{i_1,\dots,i_{d-1}})\mu_1((c_{d,i_d-1}, c_{d,i_d}])}, \end{aligned}$$

where μ_1 is the Lebesgue measure defined for the 1-dimensional sample space. For such i_d, by the definition of $\hat{F}_{l+1}$ and $\hat{\mathbf{G}}_{l+1}$,

$$\mu_{d-1}(B_{d-1}) = \hat{F}_{l+1}(\hat{\mathbf{G}}^{-1}_{l+1}(B_{d-1})) = \frac{a^{(l)}_{i_1,\dots,i_{d-1},l+1}}{C_{l+1}} \frac{\mu_{d-1}(\hat{\mathbf{G}}^{-1}_{l+1}(B_{d-1}))}{\mu_{d-1}(\hat{A}^{(l)}_{i_1,\dots,i_{d-1}})},$$

from which we obtain

$$F_{l+1}(B_{d-1} \times B_1) = C_{l+1} \frac{a^{(l)}_{i_1,\dots,i_{d-1},i_d}}{a^{(l)}_{i_1,\dots,i_{d-1},l+1}} \frac{\mu_{d-1}(B_{d-1})\mu_1(B_1)}{\mu_1((c_{d,i_d-1}, c_{d,i_d}])}$$
$$= \begin{cases} C_{l+1} \frac{\mu(B_{d-1}\times B_1)}{\mu\left((0,1]^{d-1}\times(c_{d,i_d-1},c_{d,i_d}]\right)} & (i_d = l+1), \\ a^{(l+1)}_{i_1,\dots,i_d} \frac{\mu(B_{d-1}\times B_1)}{\mu(\tilde{A}^{(l+1)}_{i_1,\dots,i_d})} & (i_d > l+1), \end{cases} \tag{23}$$

where, for $i_d > l+1$,

$$\tilde{A}^{(l+1)}_{i_1,\dots,i_d} = \hat{\mathbf{G}}_{l+1}(\hat{A}^{(l)}_{i_1,\dots,i_d}) \times (c_{d,i_d-1}, c_{d,i_d}].$$

and

$$a^{(l+1)}_{i_1,\dots,i_d} = C_{l+1} \frac{a^{(l)}_{i_1,\dots,i_{d-1},i_d}}{a^{(l)}_{i_1,\dots,i_{d-1},l+1}} \mu_{d-1}\left(\hat{\mathbf{G}}_{l+1}(\hat{A}^{(l)}_{i_1,\dots,i_d})\right).$$

Because $\mathcal{B}((0,1]^{d-1}) \times \mathcal{B}((0,1])$ generates $\mathcal{B}((0,1]^{d-1})$, from the discussion provided above, F_{l+1} is piecewise-uniform on a partition that consists of

$$(0,1]^{d-1} \times (c_{d,i_d-1}, c_{d,i_d}] \;\; (i_d \le l+1)$$

and $\tilde{A}^{(l+1)}_{i_1,\dots,i_d}$ $(i_d > l+1)$, and this partition is denoted by $\mathcal{P}^{(l+1)}$. Note that by the definition of $\hat{\mathbf{G}}_{l+1}$, and Proposition 9, which we assume holds for $(d-1)$-dimensional cases, the image of the hyper-rectangle of $\hat{A}^{(l)}_{i_1,\dots,i_{d-1}}$ under $\hat{\mathbf{G}}_{l+1}$ and $\tilde{A}^{(l+1)}_{i_1,\dots,i_d}$ are a hyper-rectangle in the $(d-1)$-dimensional space and the d-dimensional space, respectively.

The following lemma states that the partition structure $\mathcal{P}^{(l+1)}$ has a finer partition that has the "checker-board" form, as shown in the next lemma.

Lemma 6. *Let $\{D_i\}_{i=1}^I$ is a partition of the sample space $(0,1]^d$ such that every D_i is a hyper-rectangle. Then, there are sequences $\{e_{j,i_j}\}_{i_j}$ $(j = 1, 2, \dots, d)$ such that*

$$0 = e_{j,0} < e_{j,1} < \cdots < e_{j,I_j} = 1$$

and a partition $\{E_{i_1,\dots,i_d}\}_{i_1,\dots,i_d}$ defined as

$$E_{i_1,\dots,i_d} = (e_{1,i_1-1}, e_{1,i_1}] \times \cdots \times (e_{d,i_d-1}, e_{d,i_d}]$$

such that every D_i is a finite union of elements of $\{E_{i_1,\dots,i_d}\}_{i_1,\dots,i_d}$.

Its proof is straightforward because we only need to "extend" the boundaries between the rectangles $\{D_i\}_{i=1}^I$. By applying this lemma to the partition $\mathcal{P}^{(l+1)}$, it follows that there are finite sequences $\{c^{(l+1)}_{i_j}\}_{i_j}$ $(j = 1, \dots, d-1)$ such that a checkerboard-like partition consisting of the following type of rectangles

$$A^{(l+1)}_{i_1,\dots,i_d} := (c^{(l+1)}_{1,i_1-1}, c^{(l+1)}_{1,i_1}] \times \cdots \times (c^{(l+1)}_{d-1,i_{d-1}-1}, c^{(l+1)}_{d-1,i_{d-1}}] \times (c_{d,i_d-1}, c_{d,i_d}]$$

is finer than $\mathcal{P}^{(l+1)}$. With this partition, the measure F_{l+1} is written as for $B \in \mathcal{B}((0,1]^d)$

$$F_{l+1}(B) = \sum_{i=1}^{l+1} C_i \frac{\mu(B \cap (0,1]^{d-1} \times (c_{d,i-1}, c_{d,i}])}{\mu((0,1]^{d-1} \times (c_{d,i-1}, c_{d,i}])} + \sum_{i=l+2}^{I_d} \sum_{i_1,\ldots,i_{d-1}} a^{(l)}_{i_1,\ldots,i_d} \frac{\mu(B \cap A^{(l)}_{i_1,\ldots,i_d})}{\mu(A^{(l)}_{i_1,\ldots,i_d})}.$$

Because this result holds for $l = 1, \ldots, I_d - 1$, there exists a sequence of mappings $\mathbf{G}_1, \ldots, \mathbf{G}_{I_d} \in \boldsymbol{\mathcal{G}}^{L,d}$ such that a push-forward measure $H := (\mathbf{G}_{I_d} \circ \cdots \circ \mathbf{G}_1)\#F$ has a form

$$H(B) = \sum_{i=1}^{I_d} C_i \frac{\mu(B \cap (0,1]^{d-1} \times (c_{d,i-1}, c_{d,i}])}{\mu((0,1]^{d-1} \times (c_{d,i-1}, c_{d,i}])}.$$

Define an one-dimensional probability measure $\hat{H}$ as follows

$$\hat{H}(B_1) = \sum_{i=1}^{I_d} C_i \frac{\mu_1(B_1 \cap (c_{d,i-1}, c_{d,i}])}{\mu_1((c_{d,i-1}, c_{d,i}])} \text{ for } B_1 \in \mathcal{B}((0,1]).$$

Then, by the assumption (or Proposition 8), there exists a mapping $\hat{G}_0 \in \boldsymbol{\mathcal{G}}^{2,1}$ such that $\hat{\mathbf{G}}_0\#\hat{H} = \mu_1$. With this mapping, we define another mapping $\mathbf{G}_0 : (0,1] \mapsto (0,1]$ such that for $x = (x_1, \ldots, x_d)$,

$$\mathbf{G}_0(x) = (x_1, \ldots, x_{d-1}, \hat{G}_0(x_d)).$$

This mapping moves input points only in the dth dimension according to $\hat{G}_0$ so it is written as a composition of tree CDFs defined on trees with 2 terminal node and thus an element of $\mathcal{G}^{L,d}$. Hence $\mathbf{G}_0 \in \boldsymbol{\mathcal{G}}^{L,d}$. Fix $i \in \{1, \ldots, I_d\}$. For a measurable set $B_{d-1} \times B_1$ such that

$$B_{d-1} \times B_1 \in \mathcal{B}((0,1]^{d-1}) \times \hat{G}_0((c_{d,i-1}, c_{d,i}]),$$

because H is piecewise uniform, we have

$$\begin{aligned} \mathbf{G}_0\#H(B_{d-1} \times B_1) &= H(\mathbf{G}_0^{-1}(B_{d-1} \times B_1)) = H(B_{d-1} \times \hat{\mathbf{G}}_0^{-1}(B_1)) \\ &= C_i \frac{\mu(B_{d-1} \times \hat{\mathbf{G}}_0^{-1}(B_1))}{\mu((0,1]^{d-1} \times (c_{d,i-1}, c_{d,i}])} \\ &= C_i \frac{\mu_{d-1}(B_{d-1})\mu_1(\hat{\mathbf{G}}_0^{-1}(B_1))}{\mu_1((c_{d,i-1}, c_{d,i}])}. \end{aligned}$$

On the other hand,

$$\mu_1(B_1) = \hat{H}(\hat{\mathbf{G}}_0^{-1}(B_1)) = C_1 \frac{\mu_1(\hat{\mathbf{G}}_0^{-1}(B_1))}{\mu_1((c_{d,i-1}, c_{d,i}])}.$$

Hence, we obtain

$$\mathbf{G}_0\#H(B_{d-1} \times B_1) = \mu_{d-1}(B_{d-1})\mu_1(B_1) = \mu(B_{d-1} \times B_1).$$

Therefore, we conclude that

$$\mathbf{G}_0\#H = (\mathbf{G}_0 \circ \mathbf{G}_{I_d} \circ \cdots \circ \mathbf{G}_1)\#H = \mu. \quad \square$$

The result of Proposition 9 can be described in a simplified form as in the next corollary. This proof immediately follows Proposition 9 and Lemma 6.

Corollary 1. *Let* $\{E_i\}_{i=1}^I$ *is a partition of the sample space* $(0,1]^d$ *such that* E_i *is a rectangle with a form*

$$E_i = (a_{i,1}, b_{i,1}] \times \cdots \times (a_{i,d}, b_{i,d}],$$

and F *be a piecewise uniform probability measure defined on the partition:*

$$F(B) = \sum_{i=1}^{I} \beta_i \frac{\mu(B \cap E_i)}{\mu(E_i)} \text{ for } B \in \mathcal{B}((0,1]^d),$$

where $\beta_i > 0$. *Then* $F \in \mathcal{G}^L$ *for* $L \geq d+1$.

B.4 Proof of Theorem 3

We finally provide the proof of Theorem 3, which can be obtained by adding minor modifications to the proof of Theorem 4 in Wong and Ma (2010).

Let f^* denote F^*'s density function, and we first assume that f^* is uniformly continuous. For $\epsilon > 0$, there exists $\epsilon' > 0$ such that $\log(1+\epsilon') < \epsilon$. Since the function f^* is uniformly continuous, there exists $\delta > 0$ such that

$$|x - y| < \delta \Rightarrow |f^*(x) - f^*(y)| < \epsilon'.$$

Let $\{E_i\}_{i=1}^I$ is a partition of the sample space $(0,1]^d$ such that E_i has a rectangle shape and $\text{diam}(E_i) < \delta$. Define a function $\tilde{g}$ as

$$\tilde{g} = \sum_{i=1}^{I} \left\{ \sup_{x \in E_i} f^*(x) \right\} \mathbf{1}_{E_i}(x) \text{ for } x \in (0,1]^d.$$

Let $C = \int \tilde{g} d\mu$. Because $\tilde{g}(x) \geq f^*(x)$ for $x \in (0,1]^d$, we have $C \geq 1$ and

$$\begin{aligned} 0 \leq C - 1 = \int (\tilde{g} - f^*) d\mu = \sum_{i=1}^{I} \int_{E_i} (\tilde{g}(x) - f^*(x)) d\mu \\ \leq \sum_{i=1}^{I} \int_{E_i} \epsilon' d\mu = \epsilon'. \end{aligned}$$

Define a density function $g := \tilde{g}/C$. The corresponding probability measure G is an element of $\mathcal{G}^L$ by Corollary 1. Hence, for the two measures F^* and G, we can bound the KL divergence as follows

$$\begin{aligned} KL(F||G) = \int f^* \log \frac{f^*}{g} d\mu = \int f^* \log \frac{f^*}{\tilde{g}} d\mu + \int f^* \log C d\mu \\ \leq \log C \leq \log(1+\epsilon') < \epsilon. \end{aligned}$$

We next consider the general case, where we assume $f^* \leq M$ for some $M > 0$. By Lusin's theorem, for any $\tilde{\epsilon} > 0$, there exits a closed set B such that $\mu(B^c) < \tilde{\epsilon}$ and f^* is uniformly continuous on B. Using this fact, we modify the first discussion as follows. The definition of $\tilde{g}$ is modified as follows: If $E_i \cap B \neq \emptyset$, for $x \in E_i$, we let

$$\tilde{g}(x) = \sup_{x \in E_i \cap B} f^*(x).$$

Otherwise, $g(x) = M$. With this modification, we obtain

$$0 \le C - 1 = \int (\tilde{g} - f^*) d\mu = \int_B (\tilde{g} - f^*) d\mu + \int_{B^c} (\tilde{g} - f^*) d\mu$$
$$\le \epsilon' + M\tilde{\epsilon},$$

which can be arbitrarily small, so the same result follows. □

Appendix C. Details on Learning Probability Measures with the Pólya Tree Process

This section provides details on the weak learners we use to fit tree measures to the residuals in the estimation. The algorithm is based on the PT-based method proposed in Awaya and Ma (2024), and interested readers may refer to this paper.

C.1 Theoretical Justification of Using the PT-based Model

As shown in Section Proposition 5, the improvement in the entropy loss is maximized when a fitted tree is a solution of the problem

$$\arg\max_{T \in \mathcal{T}} \sum_{A \in \mathcal{L}(T)} \tilde{F}_k^{(n)}(A) \log \frac{\tilde{F}_k^{(n)}(A)}{\mu(A)},$$

where $\tilde{F}_k^{(n)}$ is the empirical measure defined by the residuals $\boldsymbol{r}^{(k-1)} = \{r_i^{(k-1)}\}_{i=1}^n$. As $n \to \infty$, the empirical measure $\tilde{F}_k^{(n)}(B)$ converges to $\tilde{F}_k(B)$ for $B \subset (0,1]^d$, where $\tilde{F}_k$ is the true distribution of the residuals defined by the previous tree-CDFs $\mathbf{G}_1, \ldots, \mathbf{G}_{k-1}$. At this population level, the maximization problem is written as

$$\arg\max_{T \in \mathcal{T}} \sum_{A \in \mathcal{L}(T)} \tilde{F}_k(A) \log \frac{\tilde{F}_k(A)}{\mu(A)},$$

and we can show that this maximization is equivalent to minimizing the KL divergence $KL(\tilde{F}_k || \tilde{F}_k|_T)$, where $\tilde{F}_k|_T$ is "a tree-approximation of $\tilde{F}_k$ under T", namely,

$$\tilde{F}_k|_T(B) = \sum_{A \in \mathcal{L}(T)} \tilde{F}_k(A) \frac{\mu(B \cap A)}{\mu(A)} \text{ for } B \in (0,1]^d.$$

Theorem 4.1 in Awaya and Ma (2024) shows that the posterior of trees also concentrates on the minimizer of $KL(\tilde{F}_k || \tilde{F}_k|_T)$, and this result implies that at the population level, or when n is large, we can find the tree that maximizes the improvement in the entropy loss or similar ones by checking the posterior of trees.

C.2 Details on the sampling algorithm

Suppose we have obtained the residuals at the beginning of the boosting algorithm. Since the task of fitting a new measure to the residuals is essentially the same for all steps, we drop

the k, the index of the trees and measures consisting of the ensemble, from the notations for simplicity. Then the residuals are denoted by $\boldsymbol{r} = (r_1, \ldots, r_n)$, and our task at each step is to capture their distributional structure by fitting a dyadic tree. In the section, we provide details on the prior distributions introduced for the tree T and the stochastic top-down algorithm we use to find a tree with good fitting. We also describe an algorithm to obtain the tree-based probability measure we add to the ensemble given the learned tree structure, which incorporates shrinkage.

C.2.1 Prior distribution of T

As in Awaya and Ma (2024), we construct a prior of T by introducing the random splitting rule for each node A. First, we introduce the stopping variable $S(A)$ that takes 0 or 1, and if $S(A) = 1$, we stop splitting A and otherwise split A. Here we set $P(S(A) = 1)$ to 0.5. In the latter case, we next define the dimension variable $D(A)$ and the location variable $L(A)$. If $D(A) = j$ $(j = 1, \ldots, d)$, the node A is split in the jth dimension, and the location of the boundary is determined by $L(A) \in (0, 1)$, in which 0 (or 1) corresponds to the left (or right) end point. Their prior distributions are as follows:

$$\begin{aligned} P(D(A) = j) &= 1/d, \ (j = 1, \ldots, d), \\ P(L(A) = l/N_L) &= \frac{1}{N_L - 1} \ (l = 1, \ldots, N_L - 1), \end{aligned}$$

where $N_L - 1$ is the number of grid points, which is 127 in the estimation.

On the tree T, we also define a random measure $\tilde{G}$, with which we can define the likelihood of the residuals $\boldsymbol{r}$. The prior of the measure $\tilde{G}$ is defined by introducing the parameters $\theta(A) = \tilde{G}(A_l \mid A)$, where A_l is the left child node, for every non-terminal node A. They follow the prior distribution specified as

$$\theta(A) \sim Beta(\theta_0(A), 1 - \theta_0(A)), \ \theta_0(A) = \frac{\mu(A_l)}{\mu(A)}.$$

The joint model of the tree T and the measure $\tilde{G}$ can be seen as a special case of the density estimation model that is referred to as the adaptive Pólya tree (Ma, 2017) model in Awaya and Ma (2024) with the number of the latent states being 2.

We note that this random measure $\tilde{G}$ is introduced just to define the marginal posterior of T, namely, $P(T \mid \boldsymbol{r})$ and to find a tree with a large marginal posterior, which captures the distributional structures of the residuals well. This Bayesian model also could be used to obtain a new tree-based measure to add to the ensemble: for example, we can use the posterior mean of $\tilde{G}$ given the tree. In the estimation, however, we obtain the new tree-based measure with the node-specific shrinkage method introduced in Section 2.7.2 because this method is theoretically justified in terms of improving the loss (see Proposition 7) and also by the empirical results provided in Section 3.1.

The detailed algorithms to construct the tree and the tree-based measure are provided in the following sections.

C.2.2 Top-down stochastic algorithm

The particle filter proposed in Awaya and Ma (2024) is shown to be effective to sample from the posterior of trees. This original algorithm, however, has drawbacks when seen as a component of the boosting from a viewpoint of computational cost:

1. In the particle filter, we construct thousands of candidate trees, but this strategy may make the whole boosting algorithm too time-consuming since in the boosting algorithm we need to repeat fitting trees to the residuals many times.

2. In the original algorithm, we do not stop splitting nodes until we reach the bottom nodes unless the number of included observations is too small. (Technically speaking, this is because the stopping variables, or the latent variables in general, are integrated out in the sampling.) The number of nodes generated in a tree, however, tends to be large especially when the sample size is large, and constructing such large trees repeatedly in the boosting algorithm is also too time-consuming. The computation cost would become reasonable if we "give up non-promising nodes", that is, stop dividing nodes if no interesting structures are found there.

From these reasons, we modify the original algorithm as follows: (i) Instead of generating many candidate trees, we set the number of particles to one, that is to say, construct a tree by randomly splitting nodes on the tree in a top-down manner. Hence the algorithm is similar to the top-down greedy method, but in our algorithm one selects splitting rules stochastically. (ii) For each active node, we compare possible splitting rules and the decision of stopping the splitting, where the latter option is added to the algorithm. This comparison is based on their posterior probabilities, and the splitting tends to be stopped if the conditional distribution is close to uniform.

For an active node A, the possible decisions are compared based on the following quantities that are seen as "prior × marginal likelihood". A conceptually very similar algorithm for supervised learning is proposed in He and Hahn (2023). For the decision of stopping, we compute

$$L_{\emptyset} = P(S(A) = 1)\mu(A)^{-n(A)},$$

where $n(A)$ is the number of residuals included in A. On the other hand, for the splitting rule $D(A) = j$ and $L(A) = l/N_L$ that divides A into A_l and A_r, we compute

$$\begin{aligned}
L_{j,l} &= P(S(A) = 0, D(A) = j, L(A) = l/N_L) \\
&\quad \times \int Beta(\theta \mid \theta_0(A), 1 - \theta_0(A))\theta^{n(A_l)}(1-\theta)^{n(A_r)}d\theta \\
&\quad \times \mu(A_l)^{-n(A_l)}\mu(A_r)^{-n(A_r)} \\
&= P(S(A) = 0, D(A) = j, L(A) = l/N_L) \\
&\quad \times \frac{Be(\theta_0(A) + n(A_l), 1 - \theta_0(A) + n(A_r))}{Be(\theta_0(A), 1 - \theta_0(A))} \\
&\quad \times \mu(A_l)^{-n(A_l)}\mu(A_r)^{-n(A_r)},
\end{aligned}$$

where $Be(\cdot)$ is the beta function. Based on these quantities, we choose to stop the splitting with probability

$$\frac{L_\emptyset}{L_\emptyset + \sum_{j=1}^{d}\sum_{l=1}^{N_L-1} L_{j,l}.}$$

Otherwise, we choose the splitting rule $D(A) = j$ and $L(A) = l/N_L$ with probability

$$\frac{L_{j,l}}{\sum_{j'=1}^{d}\sum_{l'=1}^{N_L-1} L_{j',l'}}.$$

C.2.3 Fitting a tree measure to the residuals

This section provides an algorithm to obtain a new tree-based probability measure, denoted by G to add to the ensemble given a learned tree T in each iteration of the boosting algorithm. This measure G incorporates the shrinkage method described in Section 2.7.2.

To construct G, we just need to specify the probability assignment $G(A_l \mid A)$ for every non-terminal node A. Every time we split a node A in the top-down algorithm, we compute

$$G_k(A_l \mid A) = (1 - c(A))\mu(A_l \mid A) + c(A)\frac{n_k(A_l)}{n_k(A)},$$

where $c(A)$ is the node-specific learning rate (see Section 2.7.2 for the definition) and $n_k(A_l)$ and $n_k(A)$ are the number of residuals included in A_l and A, respectively.

Appendix D. Details of the 48-dimensional Experiments

In the experiment, we used the following three scenarios.

Scenario A: $(X_1, X_2, X_3, X_4), \dots, (X_{45}, X_{46}, X_{47}, X_{48})$ are independent, and each tuple follows

$$\text{Normal}(\boldsymbol{\mu}, \Sigma),$$

where $\boldsymbol{\mu} = (0.5, \dots, 0.5)$ and

$$\Sigma_{i,j} = 0.9^{|i-j|}/8^2,\ i, j = 1, 2, 3, 4.$$

Scenario B: $(X_1, X_2), \dots, (X_{47}, X_{48})$ are independent, and each pair follows

$$\begin{aligned}
&\frac{1}{10}\text{Beta}(x_1 \mid 1, 1) \times \text{Beta}(x_2 \mid 1, 1) + \frac{3}{10}\text{Beta}(x_1 \mid 15, 45) \times \text{Beta}(x_2 \mid 15, 45)\\
&\quad + \frac{3}{10}\text{Beta}(x_1 \mid 45, 15) \times \text{Beta}(x_2 \mid 22.5, 37.5)\\
&\quad + \frac{3}{10}\text{Beta}(x_1 \mid 37.5, 22.5) \times \text{Beta}(x_2 \mid 45, 15).
\end{aligned}$$

Scenario C: $(X_1, X_2), \dots, (X_{47}, X_{48})$ are independent, and each pair follows

$$\begin{aligned}
&\frac{1}{3}\mathbf{1}_{[0.1,0.45]\times[0.35,0.9]}(x_1, x_2) + \frac{1}{3}\mathbf{1}_{[0.2,0.8]\times[0.45,0.5]}(x_1, x_2)\\
&\quad + \frac{1}{3}\mathbf{1}_{[0.7,0.9]\times[0.05,0.6]}(x_1, x_2).
\end{aligned}$$

Appendix E. Additional Tables and Figures

(c_0, γ)	POWER	GAS	HEPMASS	MINIBOONE
$(0.1, 0.0)$	0.004	0.060	0.028	0.075
$(0.1, 0.5)$	0.004	0.023	0.032	0.064
(c_0, γ)	AReM	CASP	BANK	
$(0.1, 0.0)$	0.022	0.038	0.078	
$(0.1, 0.5)$	0.015	0.030	0.023	

Table 4: The standard deviations of the average predictive scores based on 30 different random seeds.

POWER	GAS	HEPMASS	MINIBOONE
4.5	9.6	24.3	15.3
AReM	CASP	BANK	
1.4	1.6	9.0	

Table 5: The average computation time (seconds) for simulating 10,000 observations based on 30 different random seeds. The tuning parameters c_0, γ are set to 0.1 and 0.5, respectively.

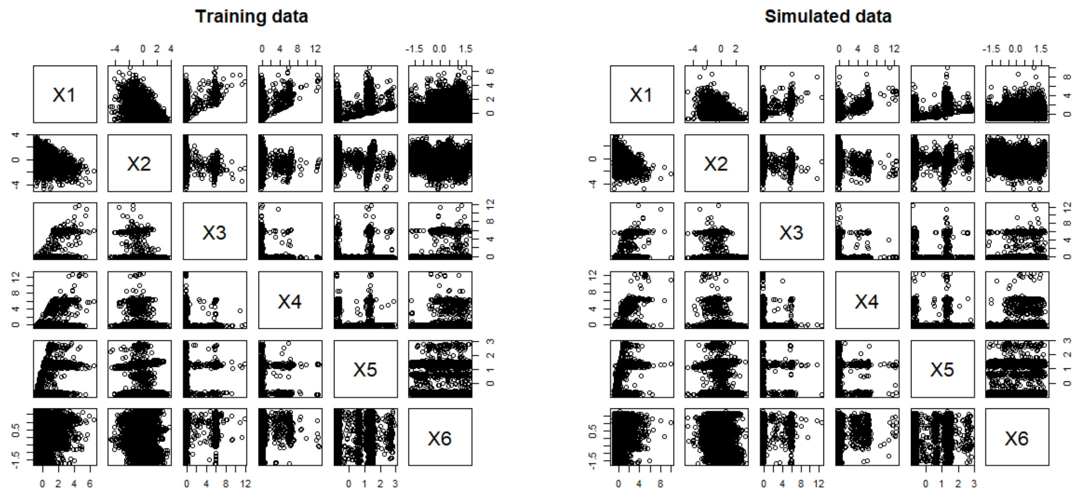

Figure 8: The training set of the POWER data (a subset of size 10,000 is visualized) and 10,000 observations simulated from the learned probability measure.

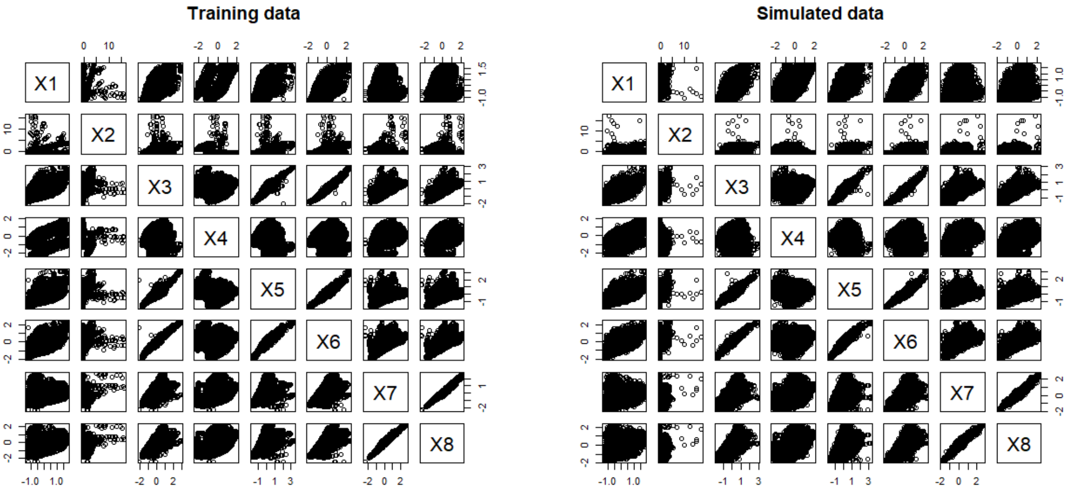

Figure 9: The training set of the GAS data (a subset of size 10,000 is visualized) and 10,000 observations simulated from the learned probability measure.

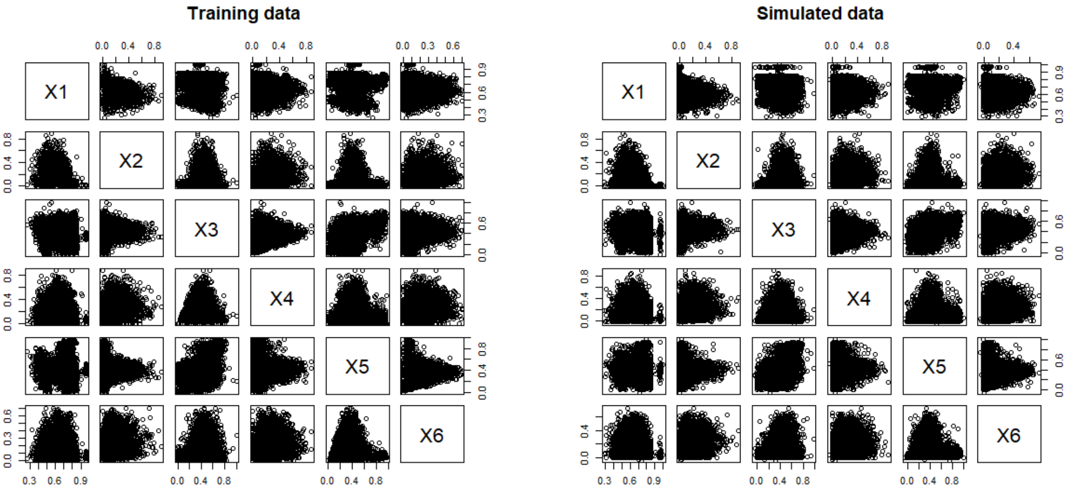

Figure 10: The training set of the AReM data (a subset of size 10,000 is visualized) and 10,000 observations simulated from the learned probability measure.
